# Non-monotonic auto-regulation in single gene circuits

**DOI:** 10.1371/journal.pone.0216089

**Published:** 2019-05-02

**Authors:** Lana Descheemaeker, Eveline Peeters, Sophie de Buyl

**Affiliations:** 1 Applied Physics Research Group, Physics Department, Vrije Universiteit Brussel, Brussels, Belgium; 2 Interuniversity Institute of Bioinformatics in Brussels, Vrije Universiteit Brussel - Université Libre de Bruxelles, Brussels, Belgium; 3 Research Group of Microbiology, Department of Bioengineering Sciences, Vrije Universiteit Brussel, Brussels, Belgium; Universitat Pompeu Fabra, SPAIN

## Abstract

We theoretically study the effects of non-monotonic response curves in genetic auto-regulation by exploring the possible dynamical behaviors for such systems. Our motivation is twofold: we aim at conceiving the simplest genetic circuits for synthetic biology and at understanding the natural auto-regulation of the LrpB protein of the *Sulfolobus solfataricus* archaeon which exhibits non-monotonicity. We analyzed three toy models, based on mass-action kinetics, with increasing complexity and sought for oscillations and (fast) bistable switching. We performed large parameter scans and sensitivity analyses, and quantified the quality of the oscillators and switches by computing relative volumes in parameter space reproducing the sought dynamical behavior. All single gene systems need finely tuned parameters in order to oscillate, but bistable switches are more robust against parameter changes. We expected non-monotonic switches to be faster than monotonic ones, however solutions combining both auto-activation and repression in the physiological range to obtain fast switches are scarce. Our analysis shows that the Ss-LrpB system can not provide a bistable switch and that robust oscillations are unlikely. Gillespie simulations suggest that the function of the natural Ss-LrpB system is sensing via a spiking behavior, which is in line with the fact that this protein has a metabolic regulatory function and binds to a ligand.

## Introduction

Synthetic biology aims at building an extended toolbox of elementary genetic circuits and efficient designs for assembling them. These building blocks are inspired by electronics. The biological equivalent of many circuits have been built for timekeeping, electronic memory storage, toggle switches, oscillators, cascades, pulse generators, time-delayed circuits, spatial patterning and logic gate behavior [1–6]. In order to construct predictable complex circuits, each building block must itself be predictable. In this work, we searched for the simplest genetic networks consisting of a single gene that produce, at the deterministic level, a dynamical behavior other than a stable steady state, i.e. oscillations or bistable switching. We also assessed the importance of molecular noise by performing Gillespie simulations. Our motivation is twofold. First, conceiving the simplest building blocks with only one gene is of interest to synthetic biology as it can potentially reduce undesired interference with other modules and facilitate the construction of complex circuits based on orthogonal compounds. Second, we want to understand the possible functions that can be fulfilled by single gene circuits. We are in particular interested in the function of the protein Ss-LrpB in the archaeon *Sulfolobus solfataricus*, both in the natural context and for its potential utility to develop simple building blocks for synthetic biology with Archaea, a territory almost unexplored. The relevance of non-monotonic regulation is broader than the Ss-LrpB system and is of importance for instance for toxin-antitoxin systems [7, 8].

Ss-LrpB is a protein forming dimers that regulates positively or negatively its own production via binding to three sites in front of the promoter. This system can be considered as a one gene mixed feedback loop: at low concentrations the protein activates itself, and at high concentrations it represses its own production [9]. We wonder what could be the role of such a complicated gene architecture, and under which circumstances this non-monotonic auto-regulation generates oscillations, bistability, bursting behavior or simply leads to a steady state. Since the Ss-LrpB protein has a metabolic regulatory function and binds to a ligand, we formulated two hypothetic dynamical behaviors relevant for sensing. Oscillations can provide a sensing mechanism which measures the input signal at regular time intervals. Alternatively, bistability of this protein could provide a switch to maintain a high concentration of Ss-LrpB when the ligand is present (absent) and low concentration of the protein otherwise.

**Fig 1:**
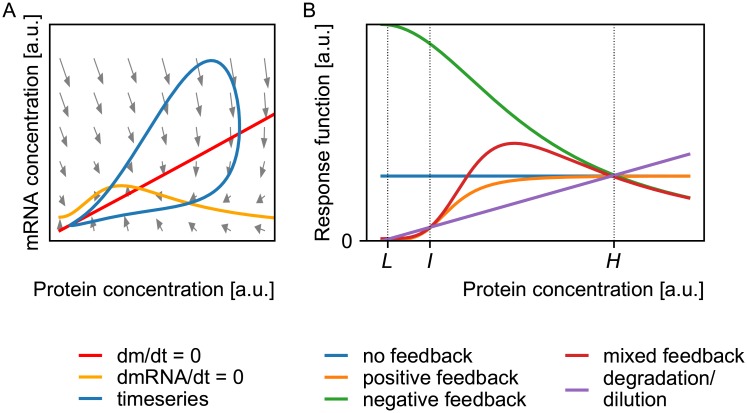
Conceptual representation of the two hypotheses. (A) Oscillations: a non-monotonic response curve, together with implicit delay can give rise to oscillatory behavior. A non-monotonic response, i.e. the mRNA production is non-monotonic with respect to the protein concentration (*m*), results in a non-monotonic nullcline (dmRNA/dt = 0). With sufficient delay (implicit or explicit), the steady state which is the intersection of both nullclines (dm/dt = 0 and dmRNA/dt = 0) becomes unstable and the time series becomes a clockwise oscillation in the phase plane. The grey arrows denote local velocities under the assumption of quasi steady state for the remaining variables, i.e. the DNA states and dimer concentration. (B) A fast bistable switch: Steady state is obtained when the production rate (feedback) and removal rate (degradation/dilution) are equal. For concentrations where the feedback is bigger than the degradation/dilution, the concentration will increase, for concentrations where the feedback is smaller than the degradation/dilution, the concentration will decrease. Positive feedback can lead to bistability, i.e. there is a low (*L*) and high stable steady state (*H*), the intermediate state (*I*) is unstable. Because the instantaneous speed of the reaction is the difference of the response function and the degradation/dilution, the induction time to the steady state (*H*) will be smaller for systems with negative feedback than for systems with no feedback. Similarly, mixed feedback can speed up the induction time of a bistable switch, i.e. the time it takes to go from the unstable intermediate state *I* to the stable high steady state *H*.

It is well known that bistability can be obtained through auto-activation, while auto-repression is known to speed up the reaction time [10]. We hypothesize that the dual feedback can result in a faster switch, see Fig 1B. In order to go from one steady state to the other an external trigger needs to decrease/increase the concentration past the intermediate unstable state. Although this switch is reversible, the main advantage of the mixed feedback is the time gain to switch from the intermediate state to the high steady state. Fast switching provides increased fitness at the individual level, contrarily to the bet-hedging strategy operating at the population level. Notice that bet-hedging also relies on a bistable switch, however the switching is stochastic [11]. Another hypothesis for the Ss-LrpB dynamics is related to the possibility that the threshold concentration of the protein needed to sense the presence of the ligand is too high to be maintained in steady-state. Oscillatory dynamics or irregular spiking can produce a high enough concentration only at certain time intervals, thereby possibly reducing the burden on the cell. Noise could also be useful to facilitate the evolution of gene regulation [12]. Like negative feedback, non-monotonic mixed feedback can give rise to oscillations when combined with implicit delay (Fig 1A).

The broader question we addressed in this work is: what are the possible dynamical behaviors for single gene circuits? More specifically, we considered deterministic models consisting of ordinary differential equations (ODEs) based on mass-action kinetics and without explicit time delays, similarly to [13] where slow DNA unbinding kinetics promotes oscillations. Introducing explicit time delays can lead to oscillations or even chaotic behavior, as is the case for Mackey-Glass systems [14]. However they are not considered here because we are mainly interested in prokaryotes. For these organisms, time delays necessary to generate oscillations are typically 5 to 20 minutes which is considerably longer than the time to transcribe and translate a gene, even if in some cases delay-induced degrade-and-fire oscillations can be obtained for delays as short as 3 to 5 minutes [15]. The Ss-LrpB protein which inspired this work is a small protein and is expected to be produced rapidly (more details in Section Dynamics of the Ss-LrpB natural system). Implicit time delays can be provided by positive feedback [16].

In order to produce nonlinearities and negative feedback, which are crucial for oscillations without explicit time delays and to obtain interesting dynamics in general, we only allowed for dimerization and multiple binding sites. We did not include phenomenologically high Hill coefficients to obtain oscillations such as in the famous Goodwin model. This single gene oscillator without explicit time delay describes oscillatory dynamics with only three variables and only one source of nonlinearity, a Hill function. The Hill coefficient needs to be considerably high (*n* > 8) in order to obtain sustained oscillations through a Hopf bifurcation [17]. Similar limit-cycle oscillations can be obtained by fast phosphorylation and dephosphorylation of the protein [18].

**Fig 2:**
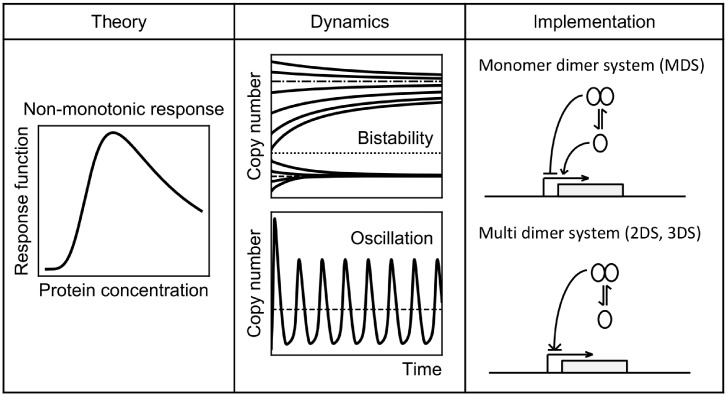
Theory, dynamics and implementation of non-monotonic response curves. Non-monotonic response curves with a maximum can lead to different types of dynamics such as bistability and oscillations. The most simple one gene implementations for such a non-monotonic response are the monomer dimer system (MDS) and multi dimer systems (details in Materials and methods).

To seek for minimal requirements to generate oscillations or a fast bistable switch based on a single gene with multiple binding sites and dimerization only, we considered toy models of increasing complexity. We first considered a single binding site. In that case, a monomer is known to be insufficient to generate oscillations without explicit delay, we therefore allowed for dimerization. The protein can bind to the binding site in both its monomeric and dimeric form and the binding site occupancy determines the up- or down-regulation of the transcription rate. We call this system the monomer dimer system (MDS). This network was based on the theoretical monomer dimer oscillator of which van Dorp discovered its oscillatory potential [19]. He considered the case of transcriptional repression by the dimer and activation by the monomer, which is a simple conceptual analog of the complete Ss-LrpB system as it is based on positive and negative feedback provided by a single gene. To our knowledge, this monomer dimer oscillator has not yet been observed in nature. We explored next the two-dimer (2DS) model which can be regulated only in dimeric form but by binding to two separate binding sites. If auto-activation occurs when one dimer is bound and auto-repression when two dimers are bound, we again obtain a simplification of the three binding site Ss-LrpB system. For prokaryotic transcription factors, dimers are generally more stable than their respective monomers. Therefore we expect that a synthetic implementation of this regulatory network in prokaryotes will be easier than an implementation of the monomer dimer system. Finally, we turned to systems with three binding sites with regulations inspired by the Ss-LrpB system (3DS) and analyzed their ability to generate oscillations, bursty behavior, or function as bistable switches. Fig 2 illustrates the questions we addressed together with our strategy to tackle them.

The paper is organized as follows, we first present our toys models, the MDS, 2DS and 3DS. We then explain our search strategy for oscillators and bistable switches. The results of these explorations in high dimensional parameter spaces are presented for each toy model, first on the oscillatory dynamics and then on the ability to serve as a fast bistable switch. We conclude by a discussion about the natural Ss-LrpB system, both at the deterministic and stochastic level.

## Materials and methods

### Toy models

We considered three families of toy models of increasing complexity which are based on mass-action kinetics. A set of ODEs describes the time derivatives of the concentrations of the different variables (DNA, mRNA and proteins). These concentrations are expressed in number per cell (instead of mol per liter), but can be fractional, for example a concentration of 1 nm in a cell of 4 fL corresponds to 2.4 molecules per cell. Expression in number per cell facilitates the comparison of deterministic and stochastic simulations. An overview of the physiological ranges of the parameters we selected based on literature for our models is provided in Table B in S1 Appendix. All python codes used in this work are available on github.

#### Monomer dimer system (MDS)

The MDS is a single gene system consisting of a protein that can regulate its production via one binding site in the regulatory region, both in its monomeric and dimeric versions. When the transcriptional fold changes *f*_*i*_ (either activating, *f*_*i*_ > 1, or repressing *f*_*i*_ < 1) of DNA bound by a monomer or dimer(s) are suitably chosen, a non-monotonic response curve can be obtained. Using mass-action kinetics, this system can be described by a deterministic model of five ODEs and 13 parameters (we refer to S1 Appendix for details, and a list of parameters is given in Table 1). It was first discovered by van Dorp that the MDS can oscillate [19]. However no wide search for oscillating parameter regions had been performed.

**Table 1:** Overview of all variables and all parameters of the different models.

variable	dimension	
DNA_i_	number per cell	concentration of DNA with no bound proteins (*i* = 0), one bound monomer (*i* = *m*), one bound dimer (*i* = *d*) or dimers bound to site(s) i (*i* ∈ {1, 2, 3})
mRNA	number per cell	mRNA concentration
*m*	number per cell	monomer concentration
*d*	number per cell	dimer concentration
*k*_*bi*_	min^−1^	binding rate of monomer (*i* = *m*), dimer (*i* = *d*) or dimer to site i (*i* ∈ {1, 2, 3})
*k*_*ui*_	min^−1^	unbinding rate of monomer (*i* = *m*), dimer (*i* = *d*) or dimer to site i (*i* ∈ 1, 2, 3)
*K*_*i*_	min^−1^	binding constant of monomer (*i* = *m*), dimer (*i* = *d*) or dimer to site i (*i* ∈ 1, 2, 3), *K*_*i*_ = *k*_*bi*_/*k*_*ui*_
*ϕ*_0_	min^−1^	transcription rate
*f*_*i*_	n.a.	transcriptional fold change when monomer (*i* = *m*) or dimer (*i* = *d*) is bound or dimers are bound to site(s) *i* (*i* ∈ {1, 2, 3}) with respect to no protein bound to the DNA
*β*	min^−1^	translation rate
*γ*_*i*_	min^−1^	degradation rate of monomer (*i* = *m*), dimer (*i* = *d*) or mRNA (*i* = mRNA)
*α*_ass_	min^−1^	association rate of monomers to dimers
*α*_diss_	min^−1^	dissociation rate of dimers to monomers
co_*b*,*uij*(*k*)_	n.a.	cooperativity factor for binding (*b*) or unbinding (*u*) between sites *i* and *j* (and *k*) (*i*, *j*, *k* ∈ {1, 2, 3})
*ω*_*ij*(*k*)_	n.a.	cooperativity factor between sites *i* and *j* (and *k*) (*i*, *j*, *k* ∈ {1, 2, 3}) *ω*_*ij*(*k*)_ = co_*bij*(*k*)_/co_*uij*(*k*)_

#### Two dimer system (2DS)

The two dimer system (2DS) we propose is again a self-regulatory gene, i.e. the gene transcribes for a protein which in dimer-form is the transcription factor of this gene. The regulatory region of the gene contains two binding sites for the transcription factor. When choosing adequately the activation/repression folds for all DNA configurations, non-monotonic curves can be obtained. A possible design to implement a promoter with a non-monotonic response synthetically is the following: when placing the first binding site for an activating transcription factor before and one binding site after the initiation site for transcription the former will activate transcription and the latter will repress transcription by steric hindrance. The deterministic model of the 2DS is slightly larger than the MDS model and has six ODEs and 16 parameters (details in Section A.2 of S1 Appendix).

#### Three dimer system (3DS)

The last system we considered is the three dimer system (3DS), a three binding site version of the 2DS. This system can describe Ss-LrpB auto-regulation without modeling the DNA loop. It is described by ten ODEs and 28 parameters (details in Section A.3 of S1 Appendix).

### (Non-)monotonicity types

**Fig 3:**
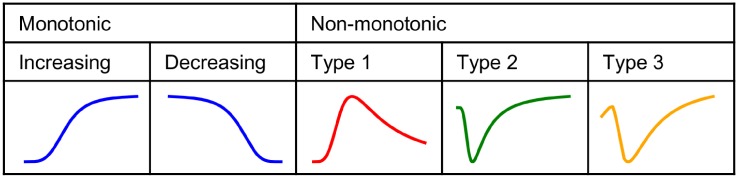
Classification of (non-)monotonic curves.

To analyze our results we made a division between different types of (non-)monotonicity. Response curves can be classified into monotonic and non-monotonic curves. We furthermore divided the non-monotic class in curves with one maximum (type 1), curves with one minimum (type 2) and curves with both a minimum and a maximum (type 3), see Fig 3.

### Search for single gene oscillators

To generate oscillations, the following four requirements need to be met: negative feedback, nonlinearity, proper balance of timescales and time delay [20]. The latter can be inserted explicitly in the model to account for instance for the time of transcription, translation, splicing, transportation between nucleus and cytoplasm or implicitly by modeling intermediate states such as phosphorylation processes or via positive feedback. Although explicit transcriptional delay can transform stable gene networks into oscillators [21–24], we focused as mentioned above on single gene oscillators that generate oscillations *in the absence of explicit time delay*.

Given the large number of parameters in all our models, we performed a random search through parameter space to seek for oscillations. We focused on oscillations that arise through a *Hopf bifurcation*. In practice, we look for systems which have one fixed point whose eigenvalues have a nonzero imaginary part and a positive real part while all other fixed points have eigenvalues with negative real parts. We do not consider more complex solutions with multiple fixed points whose eigenvalues have a positive real part, which results in the coexistence of both a locally stable steady state and locally stable oscillations. Subsequently, we imposed physical selection criteria. Given the maximum value max(*x*) and the amplitude *A*(*x*) = max(*x*) − min(*x*) of the oscillation of variable *x* (mRNA for the mRNA copy number, m for the monomer copy number and d for the dimer copy number), we require that

$$\begin{array}{cccccc} & \max(\text{mRNA})<20, & & A(\text{mRNA})>1, & & A(\text{mRNA})>\max(\text{mRNA})/3,\\ & \max(m)<5000, & & A(m)>1, & & A(m)>\max(m)/3,\\ & \max(d)<5000, & & A(d)>10\;\text{and} & & A(d)>\max(d)/3. \end{array}$$ (1)

The criteria on the maxima ensure that the copy number of mRNA remains low enough and that the copy numbers of the protein stay within their physiological bounds. For deterministic oscillations to be robust against molecular noise, we suspect that the changes in copy number of the mRNA and proteins in the deterministic model must be at least comparable to one copy number. We even set the lower limit on the amplitude to 10 for the dimer count, to obtain significant oscillations. The last criterion forces the oscillation amplitudes to be high enough with respect to the maximum values in order to distinguish the oscillation from the stochastic noise.

For every solution we performed a bifurcation analysis to assess the stability of the oscillatory behavior against parameter changes. We defined a logarithmic parameter volume *V* of a solution *s*,

$$\begin{array}{c} V\left(s\right)=\prod_{p}\log_{10}(\frac{p_{r}\left(s\right)}{p_{l}\left(s\right)}), \end{array}$$ (2)

where the product is over all *p* parameters, and *p*_*l*_(*s*) and *p*_*r*_(*s*) are the left and right bifurcation points of this parameter for solution *s* (the latter are set equal to the borders of the physiological ranges when the bifurcation point exceeds these). This volume is used to estimate the coverage of parameter space of our random scan, and the relative volume taken by oscillatory solutions. The logarithm of the values is chosen over the values themselves as fold differences are more important in biology than absolute differences.

### Search for single gene bistable switches

To find bistable switches, we seek for dynamical systems with three fixed points: a low concentration stable fixed point *L*, an intermediate unstable fixed point *I* and a high concentration steady state *H*. The dynamics of a dimeric protein is regulated by four key processes: production, degradation/dilution, dimerization and dimer dissociation. In our three toy models, the time evolution of the monomer concentration is given by the following ODE, with aforementioned processes in order:

$$\begin{array}{c} \frac{\text{d}m}{\text{d}t}=\frac{\beta {\text{DNA}}_{\text{tot}}\phi_{0}f\left(m,d\right)}{\gamma_{\text{mRNA}}}-\gamma_{m}m-2\alpha_{\text{ass}}m^{2}+2\alpha_{\text{diss}}d. \end{array}$$ (3)

This equation is obtained by assuming quasi steady state for the DNA configurations, a more elaborate derivation of this equation can be found in Section D of S1 Appendix. Using the quasi-steady state approximation for the dimer concentration, $d=\frac{\alpha_{\text{ass}}}{\alpha_{\text{diss}}+\gamma_{d}}m^{2}$ and assuming the dissociation rate of the dimer is much faster than degradation rate of the dimer (*γ*_*d*_ ≪ *α*_diss_), the last two terms of Eq 3 cancel out and the time derivative of the monomer concentration is proportional to the difference of the transcription and degradation term,

$$\begin{array}{c} \frac{\text{d}m}{\text{d}t}\propto f(d(m))-\gamma m, \end{array}$$ (4)

with $\gamma =\gamma_{m}\gamma_{\text{mRNA}}\beta^{-1}{\text{DNA}}_{\text{tot}}^{-1}\phi_{0}^{-1}$. Bistability arises when three positive values exist for *m* such that *dm*/*dt* vanishes (*dm*/*dt* = 0), i.e. the feedback term *f*(*d*(*m*)) and the degradation term *γm* are equal (Fig 1B). The shape of the transcription function *f* and number of parameters involved depend on the specific system considered. Those functions are given here under together with the ranges of the grid over which we performed a parameter scan. More details can be found in Section D of S1 Appendix.

MDS In the quasi-steady state approximation, the transcription function can be written as
  $$\begin{array}{c} f_{MDS}\left(m\right)=\frac{f_{d}{K_{d}}^{*}m^{2}+f_{m}K_{m}m+1}{K_{d}^{*}m^{2}+K_{m}m+1} \end{array}$$ (5)
  with $K_{d}^{*}=K_{d}\frac{\alpha_{\text{ass}}}{\alpha_{\text{diss}}+\gamma_{d}}$. There are only four parameters in this equation. We performed a scan over these parameters in the following ranges:
  $$\begin{array}{cccc} f_{d} & :10^{-3}\to 10^{2}, & K_{d}^{*} & :10^{-10}\to 10^{3},\\ f_{m} & :10^{-3}\to 10^{2}\;\text{and} & K_{m} & :10^{-7}\to 10^{3} \end{array}$$
  with 30 values logarithmically spaced for every range.
2DS For a two dimer model the transcription function can be written as
  $$\begin{array}{c} f_{2DS}\left(d\right)=\frac{Ad^{2}+Bd+1}{Cd^{2}+Dd+1} \end{array}$$ (6)
  with
  $$\begin{array}{cccc} C & =K_{d1}K_{d2}\omega , & A & =f_{12}C,\\ D & =K_{d1}+K_{d2}\;\text{and} & B & =f_{1}K_{d1}+f_{2}K_{d2}. \end{array}$$
  We perform a scan over parameter space analogously to the MDS case but adapting ranges to the combined variables:
  $$\begin{array}{cccc} D & :2\cdot 10^{-4}\to 2\cdot 10^{4}, & B & :10^{-3}D\to 10^{2}D,\\ C & :0.05/2010^{-4}\left(D-10^{-4}\right)\to 20/0.05{\left(D/2\right)}^{2}\;\text{and} & A & :10^{-3}C\to 10^{2}C \end{array}$$
  with 20 values logarithmically spaced for every range.
3DS For a system with three binding sites, the transcription function can be written as
  $$\begin{array}{c} f_{3DS}\left(d\right)=\frac{Ad^{3}+Bd^{2}+Cd+1}{Dd^{3}+Ed^{2}+Fd+1} \end{array}$$ (7)
  with
  $$\begin{array}{cccc} D & =K_{d1}K_{d2}K_{d3}\omega_{12}\omega_{13}\omega_{23}\omega_{123} & A & =f_{123}D,\\ E & =K_{d1}K_{d2}\omega_{12}+K_{d1}K_{d3}\omega_{13}+K_{d2}K_{d3}\omega_{23} & B & =f_{12}K_{d1}K_{d2}\omega_{12}+f_{13}K_{d1}K_{d3}\omega_{13}\\ F & =K_{d1}+K_{d2}+K_{d3} & & \;\;\;\;+f_{23}K_{d2}K_{d3}\omega_{23},\\ & & C & =f_{1}K_{d1}+f_{2}K_{d2}+f_{3}K_{d3}. \end{array}$$
  and an analogous scan was performed with 8 values logarithmically spaced for every range.

When performing the scan on the grids defined above, we imposed physical constraints:

$$\begin{array}{cc} \text{(a)}\; & I-L\geq 10,\\ \text{(b)}\; & 500<H<600\;\text{and}\\ \text{(c)}\; & I<100 \end{array}$$ (8)

where constraint (a) prohibits the lower state *L* and the intermediate state *I* from lying too close together to avoid stochastic noise to switch constantly between these states, constraint (b) fixes an interval for the high steady state *H* because the induction time depends on the level of this state, and constraint (c) prohibits the intermediate state *I* from being too high.

If the system is bistable, we can calculate the time to reach the high steady state when starting from the intermediate one. In the reduced system (Eq 4), the time it takes for the system to go from the unstable intermediate *I* to the stable high steady state *H* is proportional to the following expression

$$\begin{array}{c} \tilde{\Delta t}\propto \int_{I}^{H}\frac{dm}{f(d(m))-\gamma m}. \end{array}$$ (9)

Although this is the time obtained for the reduced system using the quasi-steady state assumption for the dimer and DNA concentrations, it provides a good estimate for the time obtained from the complete system. For every set of parameters that dictate the response curve *f* in the scan, we choose the last remaining free parameter *γ* such that the approximated induction time $\tilde{\Delta t}$ is minimized. Then we compute the induction time by doing a deterministic simulation (details in Section D.2 of S1 Appendix).

## Results

### Single gene oscillators

**Fig 4:**
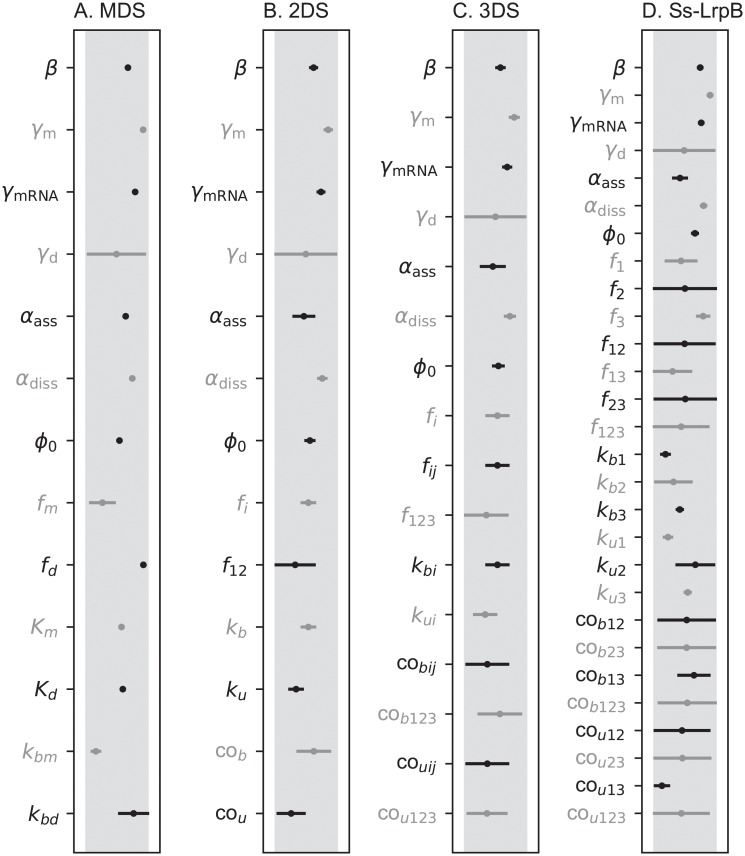
Oscillatory solutions for the different toy models. The shaded region represents the physiological range. The black and gray lines represent the mean oscillatory ranges for the different parameters. The axis is logarithmically scaled, the length of the lines thus represent fold ratios. The line for each parameter is scaled according to the physiological range of this parameter. Ranges are very small for the MDS and become wider for the 2DS and 3DS.

**Fig 5:**
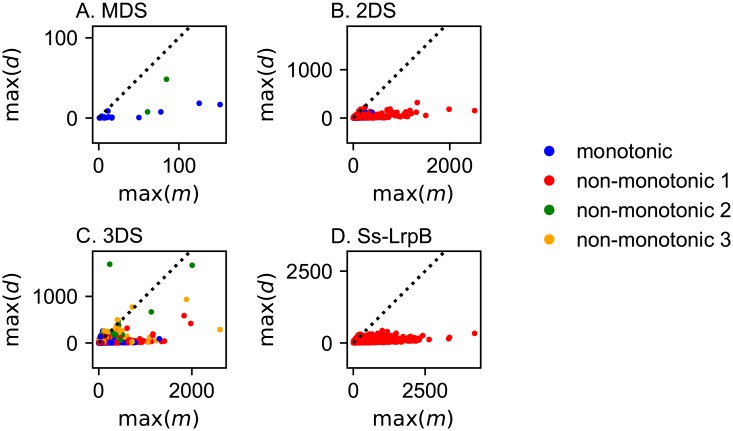
Amplitudes of oscillation in the monomer (m)—dimer (d) plane for the different toy models. The dashed line represents same copy number of monomer and dimer. Oscillations for the MDS are small: the dimer copy number does not exceed 50. The copy number of the monomer is higher than the copy number of the dimer for the majority of the solutions for all systems, contrarily to what is observed in prokaryotic systems in nature.

For the MDS very few oscillating solutions were found. Out of 2.4 ⋅ 10^8^ searched parameter sets, only 18 met the conditions for a Hopf bifurcation. Moreover, parameters of these oscillatory solutions need to be finely tuned, which is consistent with the fact that we found only very few solutions. The mean and mean logarithmic range for the different parameters are represented in Fig 4A. More detailed figures of the distribution of the ranges for the different parameters can be found in S1 Appendix. Oscillators with finely tuned parameters are not expected to be realistic due to the unavoidable noise in cellular processes. Moreover, when the system oscillates, the amplitudes of oscillations are typically very small. Amplitudes of oscillations of the monomer versus dimer are represented in Fig 5. We conclude therefore that the MDS cannot provide a realistic genetic oscillator. For the 2DS more oscillating solutions can be found. However they are still very rare. Out of the 2.4 ⋅ 10^8^ examined sets 1894 were oscillating and only 170 meet the selection criteria (Eq 1). Around 58% of the solutions are non-monotonic. Oscillatory parameter ranges are considerably wider than in the MDS case, as illustrated in Fig 4.

Even more solutions can be found for the 3DS: 2999 out of 0.8 ⋅ 10^8^ parameter sets have a Hopf bifurcation. 291 solutions remain after imposing the selection criteria. 56% of the solutions are non-monotonic. For oscillating solutions the monomer copy number is most often higher than the dimer copy number, this is the case for all toy models (Fig 5).

The results of our random scans are summarized in Table 2. To quantify the likelihood of a model to provide oscillatory solutions, we computed the logarithmic parameter volume of the scanned parameter space (definition by Eq 2), the mean volume of the found oscillatory solutions and the estimated relative volume of the found oscillatory solutions as a proxy for the capacity of the model to generate oscillations. It needs to be mentioned that the distribution of the logarithmic volumes of the different found oscillatory solutions is non-Gaussian (more details in Fig E of S1 Appendix). The ratio of the total logarithmic volume of parameter space to the mean logarithmic parameter volume of an oscillating region gives an estimate of the number of parameter sets that needs to be studied. Except for the MDS, where the amount is orders of magnitude higher than what is computationally manageable, the number of studied sets is in the same order of magnitude as this ratio. Considering the relative oscillating volumes, we conclude that it is 5 times more likely to find an oscillatory solution in the 3DS model than in the 2DS model, and 100 times more like to find an oscillatory solution in the 2DS model than in the MDS model.

**Table 2:** Summary of the search for oscillations and the scan for bistability.

		Type	MDS	2DS	3DS	SsLrpB
	Number of parameters		13	16	28	21
	Logarithmic volume of parameter space		2 ⋅ 10^7^	2 ⋅ 10^9^	10^16^	3 ⋅ 10^11^
Oscillations						
	Number of random sets		2.4 ⋅ 10^8^	2.4 ⋅ 10^8^	0.8 ⋅ 10^8^	0.8 ⋅ 10^8^
	Hopf solutions (meeting selection criteria)	M	16 (2)	789 (9)	1322 (36)	-
		N1	0 (0)	1103 (161)	973 (171)	2294 (1158)
		N2	2 (0)	2 (0)	379 (19)	-
		N3	0 (0)	0 (0)	325 (65)	-
		T	18 (2)	1894 (170)	2999 (291)	2294 (1158)
	Mean logarithmic volume of oscillating region (Eq 2)	M	5.4 ⋅ 10^−9^	4.4 ⋅ 10^0^	4.8 ⋅ 10^7^	-
		N1	-	3.1 ⋅ 10^0^	4.8 ⋅ 10^6^	8.4 ⋅ 10^2^
		N2	3.4 ⋅ 10^−10^	1.8 ⋅ 10^−3^	2.6 ⋅ 10^7^	-
		N3	-	-	2.4 ⋅ 10^6^	-
		T	4.8 ⋅ 10^−9^	3.6 ⋅ 10^0^	2.3 ⋅ 10^7^	8.4 ⋅ 10^2^
	Ratio of the total logarithmic volume of parameter space to the mean logarithmic volume of an oscillating region		5 ⋅ 10^15^	6 ⋅ 10^8^	4 ⋅ 10^8^	4 ⋅ 10^8^
	Total relative oscillating volume		7.5 ⋅ 10^−8^	7.9 ⋅ 10^−6^	3.7 ⋅ 10^−5^	2.8 ⋅ 10^−5^
Bistability						
	Number of sets		8.1 ⋅ 10^5^	1.6 ⋅ 10^5^	2.6 ⋅ 10^5^	2.6 ⋅ 10^6^
	Bistable solutions	M	4474	2334	6101	-
		N1	0	0	789	0
		N2	76	1195	2336	-
		N3	0	0	434	-
		T	4550	3529	9660	0
	Total relative bistable volume		5.6 ⋅ 10^−3^	2.2 ⋅ 10^−2^	3.7 ⋅ 10^−2^	0

M: monotonic, N1: non-monotonic type 1, N2: non-monotonic type 2, N3: non-monotonic type 3, T: total (M + N1 + N2 + N3)

### Bistable switches

**Fig 6:**
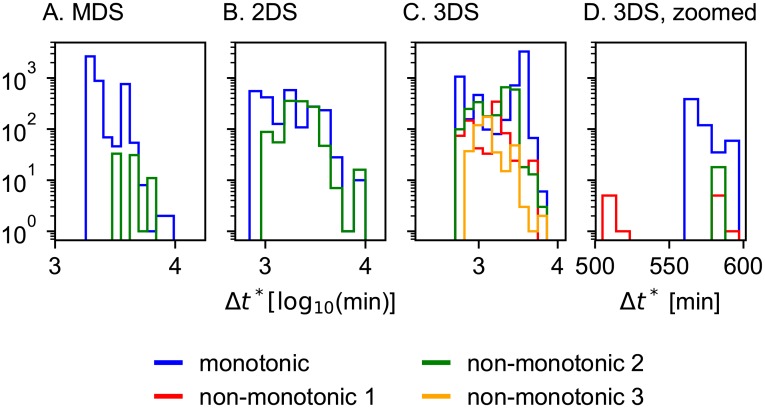
Distribution of induction times for the different toy models. For the MDS and 2DS no bistable non-monotonic type 1 solutions were found. For the 3DS many bistable solutions are found and the lowest inductions times are found for non-monotonic type 1 as we expected (panel D). Non-monotonic responses are not in general faster than monotonic responses (panel C): the induction time depends on the actual shape of the response curve and therefore on the parameters.

Our results show that the typical bistable switches are monotonically increasing or non-monotonic of type 2 for the MDS and 2DS, while for the 3DS all types of (non-)monotonicity are possible (Table 2). In Fig G in S1 Appendix the regions within parameter space providing bistable switches for the different systems are represented. For 2DS and 3DS, a large proportion of parameter space leads to bistability while the MDS needs to be finely tuned to provide this dynamical property. To assess the potential of the different models to provide bistable switches, we represented the relative logarithmic parameter volume in parameter space which they occupy (Table 2). The proportion of parameter space occupied by bistable switches clearly increases considerably from MDS to 2DS and from 2DS to 3DS. The complexity of the model increases its ability to provide switches. We also investigated the type of non-monotonicity favoring bistability. For the simpler MDS and 2DS, bistability can be obtained more easily with a monotonic or non-monotonic type 2 response curve, for the more complex 3DS, other non-monotonic types can also lead to bistability. Performing bifurcation analysis also shows that the parameter ranges providing bistability becomes larger for 3DS with respect to 2DS and MDS (details in Section D.3 of S1 Appendix). The fastest responses are found for non-monotonic type 1 curves as expected (Fig 6D), but only a small selection of the solutions is faster than any monotonic curve. The speed advantage of the non-monotonicity is thus only effective when in the most optimized case. For most of the non-monotonic solutions, a monotonic solution can be found that is as fast as the considered non-monotonic one (Fig 6C).

## Discussion about the dynamics of the Ss-LrpB natural system

**Fig 7:**
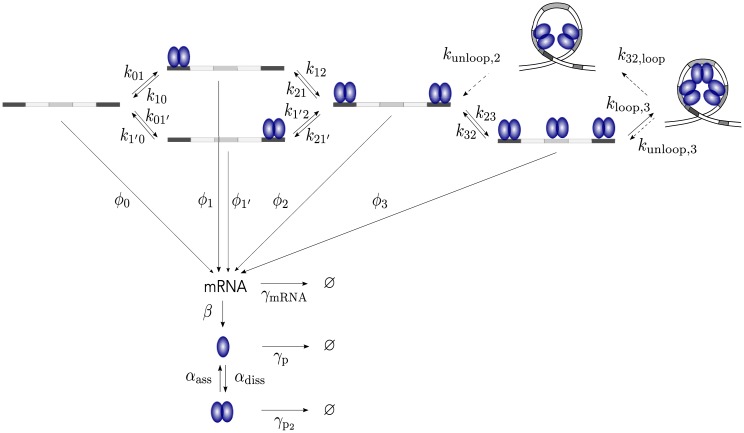
Schematic of the Ss-LrpB system. Ss-LrpB is a dimeric auto-regulative transcription factor. The control region of its own gene contains three binding sites of which the first and third one have a high affinity. Due to cooperativity the middle site will get bound and the DNA subsequently loops on itself. The system of unbinding is unknown and two hypotheses, either through direct unlooping or either by first unbinding the protein of the middle site, are denoted by dashed arrows. All DNA configurations have their own transcription rates, which lead to a non-monotonic response curve. mRNA will be translated to monomers which can dimerize. The model also takes into account degradation/dilution of all proteins (mRNA, monomers and dimers).

We conclude by a discussion on the possible dynamical behavior of the leucine responsive protein B of the archaeon *Sulfolobus solfataricus* (Ss-LrpB). This protein regulates itself in a unique way. The regulation site of this protein contains three binding sites to which the protein can bind in dimeric form. The outer sites of this regulation site have the highest affinity and will be occupied before the middle site [25]. Experimental results suggest that transcription is activated when one or both outer sites are occupied. Due to cooperativity the middle site gets bound when the outer sites are occupied and subsequently, DNA undergoes a conformational change and loops on itself. In this configuration the transcription is repressed [9]. The mechanism of unlooping is unknown. Possible manners include unlooping when the proteins in the loop are degraded [26] or via faster direct unlooping [27]. Both paths are denoted by dashed arrows in Fig 7. The temporal evolution of this system has not yet been observed experimentally, and most parameters have not been measured. As mentioned above, one hypothesis is that the Ss-LrpB system is a (possibly bursty) oscillator.

This system contains all necessary elements for oscillations: (1) negative feedback loop by the auto-repression in the state where all dimers are bound, (2) nonlinearities are provided by dimerization, cooperative binding [28], and (3) delay by modeling the intermediate states of the DNA. The sequence of gene Ss-LrpB is only 468nt long (data from KEGG library [29–31]) and with a transcription rate of minimum 40-80nt/s [32], the transcriptional delay would only be in the order of a couple of seconds. Translation goes equally fast at a rate of at least 20aa/s [32]. Because the Ss-LrpB protein only contains 155aa (data from KEGG library [29–31]) and transcription and translation are coupled in prokaryotes, the total delay due to transcription and translation remains in the order of several seconds. This motivated us to not include an explicit time delay. Furthermore, it was put forward by Novak and Tyson [20] that positive feedback can be used to add implicit delay to a negative-feedback system. The fourth element –balanced time-scales– can be obtained by fine tuning the parameters.

To find oscillatory behavior, we performed the same analysis for the Ss-LrpB system as for the 3DSs but with parameters fixed to their experimental values when known (see Section E of S1 Appendix). Furthermore it is imposed that the response is non-monotonic of type 1 and that the maximum of the response curve is at least twice the basal response and the minimum at most half the basal rate in accordance to the experimental response curve [9]. We found that some parameters, such as the degradation rates *γ*_*m*_ and *γ*_mRNA_, need to be finely tuned for oscillations. The sensitivity of each parameter is represented in Fig 4. And, as for general 3DS, many solutions have higher monomer copy numbers than dimer copy numbers (Fig 5). We conclude that the relative volume in parameter space leading to an oscillatory behavior is very small, 2.8 10^−5^. It is therefore highly unlikely that the natural Ss-LrpB system shows oscillatory behavior.

**Fig 8:**
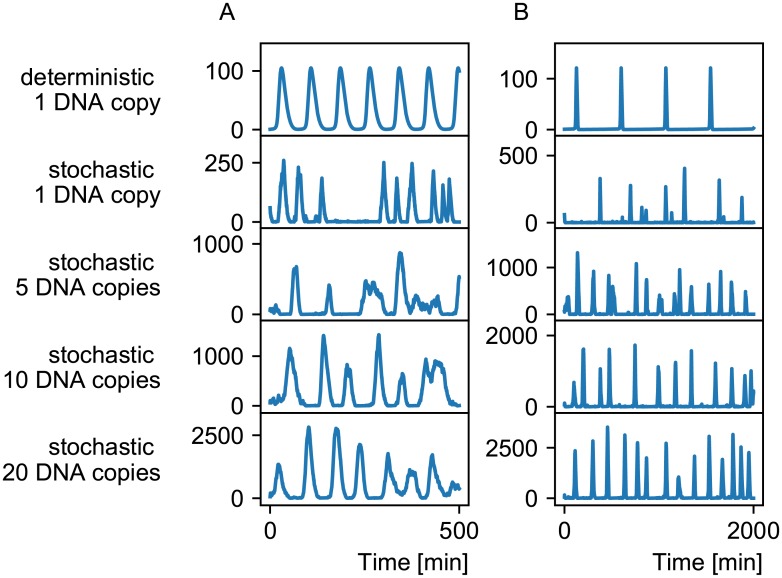
Stochastic time series. Time series of two examples of Ss-LrpB compatible systems are shown (A and B), in a deterministic and stochastic models from 1 to 20 DNA copy numbers. The oscillations become more regular for higher copy numbers. Note that the frequency is higher in the stochastic models with respect to the deterministic model for the example on the right.

To assess the role of noise in oscillatory systems, we selected two solutions which are compatible with the measured response curve of Ss-LrpB and performed stochastic simulations. Time series for different copy numbers of the DNA are shown in Fig 8. Stochastic models can differ much from the deterministic behavior, especially when considering a wild type *Sulfolobus solfataricus* cell which only contains at most two copies of the genome. In synthetic biology, target genes can be inserted in vector plasmids which can be injected in the cell in higher numbers. This would reduce the stochasticity in mRNA and protein concentrations (Fig 8).

**Fig 9:**
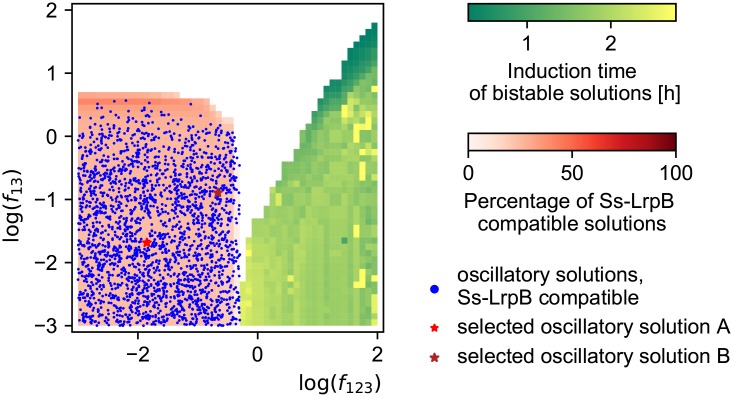
Bistable region with induction times in the *f*_123_-*f*_13_-plane. The bistable region is shown in yellow and green where the color represents the induction time of the fastest solution found for every *f*_13_-*f*_123_-combination. In red the percentages of Ss-LrpB compatible solutions are given. The regions of bistable systems (yellow-green) and Ss-LrpB compatible solutions (red) are not overlapping, we can therefore conclude that the natural Ss-LrpB system cannot exhibit bistability.

We next envisaged the possibility to have a bistable switch. We undertook the same analysis as for the 3DS bistable switch analysis but again with fewer free parameters. For the Ss-LrpB system, seven parameters have been measured and we estimated two more based on literature, as detailed in Section E of S1 Appendix. Five free parameters remain: *f*_1_, *f*_2_, *f*_3_, *f*_12_, *f*_23_ over which we performed a scan with the different *f* ranging from 10^−3^ to 10^2^. The bistable region in the *f*_13_-*f*_123_-plane is shown in yellow and green in Fig 9. Outside this region, no bistable systems were found. The color represents the induction time of the fastest solution found for each *f*_13_-*f*_123_-combination. The smallest induction times can be found when both the DNA_13_ and the DNA_123_ are activating (*f*_13_ > 1 and *f*_123_ > 1) and the response curve is monotonically increasing. In red the percentages of Ss-LrpB compatible solutions are given, i.e. solutions with response curves of non-monotonicity type 1 and a maximal transcription rate of at least twice the basal rate and a minimal rate lower than half the basal rate. Such response curves are found when DNA_123_ is repressing (*f*_123_ < 1) and DNA_13_ either somewhat activating or repressing. The regions of bistable systems (yellow-green) and Ss-LrpB compatible solutions (red) are not overlapping, we can therefore conclude that the natural Ss-LrpB system cannot exhibit bistability.

**Fig 10:**
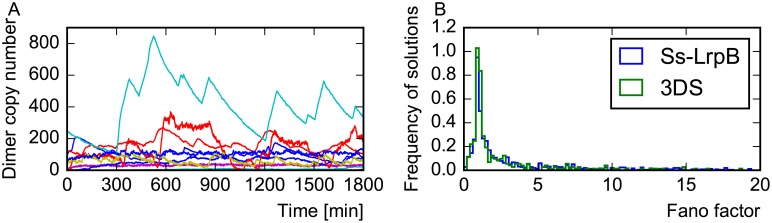
Bursting behavior of the 3DS systems. (A) 15 time traces compatible with the Ss-LrpB system (B) Histogram of Fano factors distributions for 3DS and 3DS systems compatible with Ss-LrpB systems.

We conclude that it is difficult to obtain deterministic solutions other than a stable steady state with the architecture and parameters of Ss-LrpB. Since a stable steady state can be obtained by the simplest self-regulations, we speculate that the choice for the complicated architecture of the natural Ss-LrpB system maybe roots in its stochastic properties. To test whether the natural system has evolved toward parameters leading to a noisier, and more spiky behavior, we ran stochastic timeseries for random 3DS configurations as well as for random Ss-LrpB compatible solutions. After comparison of the Fano factors, a measure of stochastic spiking, of the different sets, we concluded that the distribution of Fano factors is similar for the Ss-LrpB system and the 3DS, as illustrated in Fig 10. Since the shape of the Fano factor histograms will be affected by fixing additional values for the parameters, we cannot conclude on our hypothesis. Without extra experimental measurements, it would be time intensive to obtain a better comparison of the 3DS and the SsLrpB compatible systems. We hypothesize that the function of spiky dynamics, besides the ones proposed in [33], could be a sensing mechanism with a reduced burden on the cell. The idea is that the time averaged mean concentration of the protein is lower than the threshold concentration for sensing. Although it is slightly more frequent to exhibit rich dynamical behavior for systems with three binding sites than with two, two and three binding sites systems essentially allow for similar dynamical behaviors. We therefore expect that additional properties of systems with three binding sites, such as looping of DNA when three dimers are bound, should explain their functional role. However, without experimental evidences for a specific property, we did not elaborate more on this possibility.

## Conclusion

The question we addressed is the function of non-monotonic auto-regulation. A system with such auto-regulation exists in nature and we therefore assume it should have an advantage over other gene regulatory networks. We seek whether the non-monotonicity could result in dynamics other than a stable steady state, in particular oscillations and bistability. We studied three different single gene networks with increasing complexity that can exhibit non-monotonicity.

Despite the fact that oscillations through Hopf bifurcations become more abundant when the number of binding sites increases, they stay very scarce in parameter space. Moreover the oscillating range is finely tuned for multiple parameters. We also found that the amplitudes of monomers often exceed the amplitudes of the dimers even though the dimer is typically the active form of the protein. Because of the counterintuitive amplitudes and the finely tuned parameters, we conclude that it is highly unlikely that a natural system with a non-monotonic self response would be oscillating.

Besides oscillations, a non-monotonic type 1 response can, just as a monotonically increasing response, give rise to bistability. Repression for high concentrations can theoretically shorten the induction time to go from the unstable intermediate state to the high steady state. If the high steady state is a response on external stress, the fast reaction would give an advantage for survival for each individual cell in contrast to the stochastic switching of the bet-hedging strategy which assures survival on population level. We find indeed that the fastest solutions have a non-monotonic response type 1 curve, but only a small fraction of the non-monotonic curves is faster than monotonic implementations. The parameters dictating the response curve need to be adjusted carefully in order to be faster than monotonic responses.

For synthetic circuits, we would advise to build a fast bistable switch out of the two or three binding sites models. To build an oscillator, our sensitivity analysis provides a list of parameters that should be finely tuned and should be screened over to find a working system, however we expect it to be challenging to succeed in that enterprise.

The question of the function of the natural Ss-LrpB system is discussed in the previous section. To sum up, a scan over parameter space revealed that the response curves compatible with the natural Ss-LrpB system cannot give rise to bistability. Oscillations would need finely tuned parameters. Ruling out switches and oscillators, we consider simply steady states and fluctuations due to intrinsic noise around these steady states. We concluded by proposing that the natural Ss-LrpB system dynamics has spiking behavior around a steady state. A possible explanation for such behavior is that the concentration of Ss-LrpB needed for sensing is too high to be maintained at steady state.

## Supporting information

S1 Appendix Supporting information. Differential equations for the three models considered, physiological ranges of the parameters, details on the simulations for the induction times in bistable systems, detailed oscillatory ranges and Ss-LrpB parameters.
(PDF)

## References

[1] Stanton BC, Nielsen AAK, Tamsir A, Clancy K, Peterson T, Voigt CA. Genomic mining of prokaryotic repressors for orthogonal logic gates. Nature Chemical Biology. 2014;10(2):99–105. 10.1038/nchembio.1411 24316737 PMC4165527

[2] Mukherji S, van Oudenaarden A. Synthetic biology: understanding biological design from synthetic circuits. Nature Reviews Genetics. 2009;. 10.1038/nrg2697 19898500 PMC3138802

[3] Purnick PEM, Weiss R. The second wave of synthetic biology: from modules to systems. Nature Reviews Molecular Cell Biology. 2009;10(6):410–422. 10.1038/nrm2698 19461664

[4] Khalil AS, Collins JJ. Synthetic biology: applications come of age. Nature Reviews Genetics. 2010;11(5):367–379. 10.1038/nrg2775 20395970 PMC2896386

[5] Cameron DE, Bashor CJ, Collins JJ. A brief history of synthetic biology. Nature Reviews Microbiology. 2014;12(5):381–390. 10.1038/nrmicro3239 24686414

[6] Guet CC, Elowitz MB, Hsing W, Leibler S. Combinatorial Synthesis of Genetic Networks. Science. 2002;296(5572):1466–1470. 10.1126/science.1067407 12029133

[7] Cataudella I, Sneppen K, Gerdes K, Mitarai N. Conditional Cooperativity of Toxin—Antitoxin Regulation Can Mediate Bistability between Growth and Dormancy. PLoS Computational Biology. 2013;9(8):e1003174 10.1371/journal.pcbi.1003174 24009488 PMC3757081

[8] Gelens L, Hill L, Vandervelde A, Danckaert J, Loris R. A General Model for Toxin-Antitoxin Module Dynamics Can Explain Persister Cell Formation in E. coli. PLoS Computational Biology. 2013;9(8):e1003190 10.1371/journal.pcbi.1003190 24009490 PMC3757116

[9] Peeters E, Peixeiro N, Sezonov G. Cis-regulatory logic in archaeal transcription. Biochemical Society Transactions. 2013;41(1):326–331. 10.1042/BST20120312 23356306

[10] Rosenfeld N, Elowitz MB, Alon U. Negative Autoregulation Speeds the Response Times of Transcription Networks. Journal of Molecular Biology. 2002;323(5):785–793. 10.1016/S0022-2836(02)00994-4 12417193

[11] Cohen D. Optimizing reproduction in a randomly varying environment. Journal of Theoretical Biology. 1966;12(1):119–129. 6015423 10.1016/0022-5193(66)90188-3

[12] Wolf L, Silander OK, van Nimwegen E. Expression noise facilitates the evolution of gene regulation. eLife. 2015;4 10.7554/eLife.05856 26080931 PMC4468965

[13] Karapetyan S, Buchler NE. Role of DNA binding sites and slow unbinding kinetics in titration-based oscillators Physical Review E. 2015;92(6). 10.1103/PhysRevE.92.062712 PMC4777296 26764732

[14] Mackey M, Glass L. Oscillation and chaos in physiological control systems. Science. 1977;197(4300):287–289. 10.1126/science.267326 267326

[15] Mather W, Bennett MR, Hasty J, Tsimring LS. Delay-Induced Degrade-and-Fire Oscillations in Small Genetic Circuits Physical Review Letters. 2009;102(6). 10.1103/PhysRevLett.102.068105 PMC2924583 19257639

[16] Alon U. An introduction to systems biology: design principles of biological circuits No. 10 in Chapman & Hall/CRC mathematical and computational biology series. Boca Raton, FL: Chapman & Hall/CRC; 2007.

[17] Griffith JS. Mathematics of cellular control processes I. Negative feedback to one gene. Journal of Theoretical Biology. 1968;20(2):202–208. 10.1016/0022-5193(68)90190-2 5727239

[18] Gonze D, Abou-Jaoudé W. The Goodwin Model: Behind the Hill Function. PLoS ONE. 2013;8(8):e69573 10.1371/journal.pone.0069573 23936338 PMC3731313

[19] van Dorp M, Lannoo B, Carlon E. Generation of oscillating gene regulatory network motifs. Physical Review E. 2013;88(1). 10.1103/PhysRevE.88.012722 23944505

[20] Novák B, Tyson JJ. Design principles of biochemical oscillators. Nature Reviews Molecular Cell Biology. 2008;9(12):981–991. 10.1038/nrm2530 18971947 PMC2796343

[21] Lewis J. Autoinhibition with transcriptional delay: a simple mechanism for the zebrafish somitogenesis oscillator. Current biology: CB. 2003;13(16):1398–1408. 10.1016/S0960-9822(03)00534-7 12932323

[22] Atkinson MR, Savageau MA, Myers JT, Ninfa AJ. Development of genetic circuitry exhibiting toggle switch or oscillatory behavior in Escherichia coli. Cell. 2003;113(5):597–607. 10.1016/S0092-8674(03)00346-5 12787501

[23] Monk NAM. Oscillatory expression of Hes1, p53, and NF-kappaB driven by transcriptional time delays. Current biology: CB. 2003;13(16):1409–1413. 10.1016/S0960-9822(03)00494-9 12932324

[24] Bratsun D, Volfson D, Tsimring LS, Hasty J. Delay-induced stochastic oscillations in gene regulation. Proceedings of the National Academy of Sciences. 2005;102(41):14593–14598. 10.1073/pnas.0503858102 PMC1253555 16199522

[25] Peeters E, van Oeffelen L, Nadal M, Forterre P, Charlier D. A thermodynamic model of the cooperative interaction between the archaeal transcription factor Ss-LrpB and its tripartite operator DNA. Gene. 2013;524(2):330–340. 10.1016/j.gene.2013.03.118 23603352

[26] Stricker J, Cookson S, Bennett MR, Mather WH, Tsimring LS, Hasty J. A fast, robust and tunable synthetic gene oscillator. Nature. 2008;456(7221):516–519. 10.1038/nature07389 18971928 PMC6791529

[27] Manzo C, Zurla C, Dunlap DD, Finzi L. The Effect of Nonspecific Binding of Lambda Repressor on DNA Looping Dynamics. Biophysical Journal. 2012;103(8):1753–1761. 10.1016/j.bpj.2012.09.006 23083719 PMC3475330

[28] Buchler NE, Gerland U, Hwa T. Nonlinear protein degradation and the function of genetic circuits. Proceedings of the National Academy of Sciences. 2005;102(27):9559–9564. 10.1073/pnas.0409553102 PMC1172234 15972813

[29] Kanehisa M, Furumichi M, Tanabe M, Sato Y, Morishima K. KEGG: new perspectives on genomes, pathways, diseases and drugs. Nucleic Acids Research. 2017;45(D1):D353–D361. 10.1093/nar/gkw1092 27899662 PMC5210567

[30] Kanehisa M, Sato Y, Kawashima M, Furumichi M, Tanabe M. KEGG as a reference resource for gene and protein annotation. Nucleic Acids Research. 2016;44(D1):D457–D462. 10.1093/nar/gkv1070 26476454 PMC4702792

[31] Kanehisa M. KEGG: Kyoto Encyclopedia of Genes and Genomes. Nucleic Acids Research. 2000;28(1):27–30. 10.1093/nar/28.1.27 10592173 PMC102409

[32] Phillips R, Kondev J, Theriot J. Physical biology of the cell. New York: Garland Science; 2009.

[33] Levine JH, Lin Y, Elowitz MB. Functional Roles of Pulsing in Genetic Circuits. Science. 2013;342(6163):1193–1200. 10.1126/science.1239999 24311681 PMC4100686

