# Supplementary material for: Non-monotonic auto-regulation in single gene circuits
Source: PLoS One. 2019 May 2;14(5):e0216089. doi: 10.1371/journal.pone.0216089 (PMC6497280; doi:10.1371/journal.pone.0216089)
Supplement: S1 Appendix — Differential equations for the three models considered, physiological ranges of the parameters, details on the simulations for the induction times in bistable systems, detailed oscillatory ranges and Ss-LrpB parameters. (PDF) [file pone.0216089.s001.pdf]

Supplemental information to
"Non-monotonic autoregulation in single gene circuits"

Lana Descheemaeker[1,2], Eveline Peeters[3], Sophie de Buyl[1,2] *

1 Applied Physics Research Group, Physics Department, Vrije Universiteit Brussel, Brussels, Belgium.
2 Interuniversity Institute of Bioinformatics in Brussels, Vrije Universiteit Brussel - Université Libre de Bruxelles, Brussels, Belgium.
3 Research Group of Microbiology, Department of Bioengineering Sciences, Vrije Universiteit Brussel, Brussels, Belgium.

* sdebuyl@vub.be

Contents

A The models: Ordinary differential equations and quasi steady states 2
- A.1 MDS . . . 2
- A.2 2DS . . . 4
- A.3 3DS . . . 5

B Physiological ranges of the parameters 6

C Oscillations 7
- C.1 Bifurcation analysis . . . 7
- C.2 Oscillatory ranges distributions . . . 7

D Bistability 7
- D.1 Quasi steady state approximation . . . 8
- D.2 Induction time of bistable systems . . . 10
- D.3 Bistability in parameter space . . . 13

E SsLrpB compatibility 14

A The models: Ordinary differential equations and quasi steady states

The systems were modeled through mass action kinetics. The models take into account protein-DNA binding and unbinding, dimerization, transcription, translation and dilution/degradation of mRNA and proteins.

A.1 MDS

The monomer-dimer system contains five ordinary differential equations (ODEs):

$$\frac{\mathrm{dDNA_0}}{\mathrm{d}t} = -\left(k_{bm}m + k_{bd}d\right)\mathrm{DNA_0} + k_{um}\mathrm{DNA_m} + k_{ud}\mathrm{DNA_d},$$

$$\frac{\mathrm{dDNA_m}}{\mathrm{d}t} = k_{bm}m\mathrm{DNA_0} - k_{um}\mathrm{DNA_m},$$

$$\frac{\mathrm{dmRNA}}{\mathrm{d}t} = \phi_0\left(\mathrm{DNA_0} + f_m\mathrm{DNA_m} + f_d\mathrm{DNA_d}\right) - \gamma_{\mathrm{mRNA}}\mathrm{mRNA},$$

$$\frac{\mathrm{d}m}{\mathrm{d}t} = -k_{bm}m\mathrm{DNA_0} + k_{um}\mathrm{DNA_m} + \beta\mathrm{mRNA} - 2\alpha_{\mathrm{ass}}m^2 + 2\alpha_{\mathrm{diss}}d - \gamma_m m \text{ and}$$

$$\frac{\mathrm{d}d}{\mathrm{d}t} = -k_{bd}d\mathrm{DNA_0} + k_{ud}\mathrm{DNA_d} + \alpha_{\mathrm{ass}}m^2 - \alpha_{\mathrm{diss}}d - \gamma_d d$$

where $\mathrm{DNA_d} = \mathrm{DNA_{tot}} - \mathrm{DNA_0} - \mathrm{DNA_m}$ because the total amount of DNA is conserved. A summary of all variables and parameters is given in Table A. In case of fast dimerization and fast (un)binding of the proteins to the DNA, the time derivatives of the DNA complexes $\mathrm{DNA_i}$ and dimer d can be put equal to zero, the quasi steady state values of these variables are then

$$\mathrm{DNA_{0,\ qss}} = \frac{\mathrm{DNA_{tot}}}{K_d d + K_m m + 1},$$

$$\mathrm{DNA_{m,\ qss}} = \mathrm{DNA_{0,\ qss}} K_m m \text{ and}$$

$$d_{\mathrm{qss}} = \frac{\alpha_{\mathrm{ass}}}{\alpha_{\mathrm{diss}} + \gamma_d} m^2.$$

Table A. Overview of all variables and all parameters of the different models.

variable	dimension	
$\mathrm{DNA_i}$	number per cell	concentration of DNA with no bound proteins ($i = 0$),
		one bound monomer ($i = m$), one bound dimer ($i = d$)
		or dimers bound to site(s) i ($i \in \{1, 2, 3\}$)
mRNA	number per cell	mRNA concentration
m	number per cell	monomer concentration
d	number per cell	dimer concentration
k_{bi}	min^{-1}	binding rate of monomer ($i = m$), dimer ($i = d$)
		or dimer to site i ($i \in \{1, 2, 3\}$)
k_{ui}	min^{-1}	unbinding rate of monomer ($i = m$), dimer ($i = d$)
		or dimer to site i ($i \in 1, 2, 3$)
K_i	min^{-1}	binding constant of monomer ($i = m$), dimer ($i = d$)
		or dimer to site i ($i \in 1, 2, 3$), $K_i = k_{bi}/k_{ui}$
ϕ_0	min^{-1}	transcription rate
f_i	n.a.	transcriptional fold change when monomer ($i = m$)
		or dimer ($i = d$) is bound
		or dimers are bound to site(s) i ($i \in \{1, 2, 3\}$)
		with respect to no protein bound to the DNA
β	min^{-1}	translation rate
γ_i	min^{-1}	degradation rate of monomer ($i = m$), dimer ($i = d$)
		or mRNA ($i = \mathrm{mRNA}$)
α_{ass}	min^{-1}	association rate of monomers to dimers
α_{diss}	min^{-1}	dissociation rate of dimers to monomers
$\mathrm{co}_{b,uij(k)}$	n.a.	cooperativity factor for binding (b) or unbinding (u)
		between sites i and j (and k) ($i, j, k \in \{1, 2, 3\}$)
$\omega_{ij(k)}$	n.a.	cooperativity factor between sites i and j (and k)
		($i, j, k \in \{1, 2, 3\}$) $\omega_{ij(k)} = \mathrm{co}_{bij(k)}/\mathrm{co}_{uij(k)}$

A.2 2DS

Analogously to the system of ODEs for the MDS, we can write down the system for the 2 dimer system which contains six ODEs

$$
\begin{aligned}
\frac{\mathrm{dDNA}_0}{\mathrm{d}t} &= -\left(k_{b1}+k_{b2}\right) d\mathrm{DNA}_0 + k_{u1}\mathrm{DNA}_1 + k_{u2}\mathrm{DNA}_2, \\
\frac{\mathrm{dDNA}_1}{\mathrm{d}t} &= k_{b1} d\mathrm{DNA}_0 - k_{u1}\mathrm{DNA}_1 + k_{u2}\mathrm{co}_{u12}\mathrm{DNA}_{12} - k_{b2}\mathrm{co}_{b12} d\mathrm{DNA}_1, \\
\frac{\mathrm{dDNA}_2}{\mathrm{d}t} &= k_{b2} d\mathrm{DNA}_0 - k_{u2}\mathrm{DNA}_2 + k_{u1}\mathrm{co}_{u12}\mathrm{DNA}_{12} - k_{b1}\mathrm{co}_{b12} d\mathrm{DNA}_2, \\
\frac{\mathrm{dmRNA}}{\mathrm{d}t} &= \phi_0\left(\mathrm{DNA}_0 + f_1\mathrm{DNA}_1 + f_2\mathrm{DNA}_2 + f_{12}\mathrm{DNA}_{12}\right) - \gamma_{\mathrm{mRNA}}\mathrm{mRNA}, \\
\frac{\mathrm{d}m}{\mathrm{d}t} &= \beta\mathrm{mRNA} - 2\alpha_{\mathrm{ass}} m^2 + 2\alpha_{\mathrm{diss}} d - \gamma_m m \text{ and} \\
\frac{\mathrm{d}d}{\mathrm{d}t} &= -\left(k_{b1}+k_{b2}\right) d\mathrm{DNA}_0 - \left(k_{b2}\mathrm{DNA}_1 + k_{b1}\mathrm{DNA}_2\right)\mathrm{co}_{b12} d \\
&\quad + k_{u1}\mathrm{DNA}_1 + k_{u2}\mathrm{DNA}_2 + \left(k_{u1}+k_{u2}\right)\mathrm{co}_{u12}\mathrm{DNA}_{12} + \alpha_{\mathrm{ass}} m^2 - \alpha_{\mathrm{diss}} d - \gamma_d d
\end{aligned}
$$

where $\mathrm{DNA}_{12} = \mathrm{DNA}_{\mathrm{tot}} - \mathrm{DNA}_0 - \mathrm{DNA}_1 - \mathrm{DNA}_2$ because the total amount of DNA is conserved. A summary of all variables and parameters is given in Table A. The quasi steady states of the DNA complexes and dimer are:

$$
\begin{aligned}
\mathrm{DNA}_{0,\,\mathrm{qss}} &= \frac{\mathrm{DNA}_{\mathrm{tot}}}{\omega_{12} K_1 K_2 d^2 + \left(K_1 + K_2\right) d + 1}, \\
\mathrm{DNA}_{1,\,\mathrm{qss}} &= \mathrm{DNA}_{0,\,\mathrm{qss}} K_1 d, \\
\mathrm{DNA}_{2,\,\mathrm{qss}} &= \mathrm{DNA}_{0,\,\mathrm{qss}} K_2 d \text{ and} \\
d_{\mathrm{qss}} &= \frac{\alpha_{\mathrm{ass}}}{\alpha_{\mathrm{diss}} + \gamma_d} m^2.
\end{aligned}
$$

A.3 3DS

The 3DS contains ten ODEs:

$$\frac{\mathrm{dDNA}_0}{\mathrm{d}t} = -\left(k_{b1}+k_{b2}+k_{b3}\right)d\mathrm{DNA}_0 + k_{u1}\mathrm{DNA}_1 + k_{u2}\mathrm{DNA}_2 + k_{u3}\mathrm{DNA}_3,$$

$$\begin{aligned}\frac{\mathrm{dDNA}_1}{\mathrm{d}t} &= k_{b1}d\mathrm{DNA}_0 - k_{u1}\mathrm{DNA}_1 + k_{u2}\mathrm{co}_{u12}\mathrm{DNA}_{12} + k_{u3}\mathrm{co}_{u13}\mathrm{DNA}_{13}\\ &\quad - \left(k_{b2}\mathrm{co}_{b12} + k_{b3}\mathrm{co}_{b13}\right)d\mathrm{DNA}_1,\end{aligned}$$

$$\begin{aligned}\frac{\mathrm{dDNA}_2}{\mathrm{d}t} &= k_{b2}d\mathrm{DNA}_0 - k_{u2}\mathrm{DNA}_2 + k_{u1}\mathrm{co}_{u12}\mathrm{DNA}_{12} + k_{u3}\mathrm{co}_{u23}\mathrm{DNA}_{23}\\ &\quad - \left(k_{b1}\mathrm{co}_{b12} + k_{b3}\mathrm{co}_{b23}\right)d\mathrm{DNA}_2,\end{aligned}$$

$$\begin{aligned}\frac{\mathrm{dDNA}_3}{\mathrm{d}t} &= k_{b3}d\mathrm{DNA}_0 - k_{u3}\mathrm{DNA}_3 + k_{u1}\mathrm{co}_{u13}\mathrm{DNA}_{13} + k_{u2}\mathrm{co}_{u23}\mathrm{DNA}_{23}\\ &\quad - \left(k_{b1}\mathrm{co}_{b13} + k_{b2}\mathrm{co}_{b23}\right)d\mathrm{DNA}_3,\end{aligned}$$

$$\begin{aligned}\frac{\mathrm{dDNA}_{12}}{\mathrm{d}t} &= k_{b1}\mathrm{co}_{b12}d\mathrm{DNA}_2 + k_{b2}\mathrm{co}_{b12}d\mathrm{DNA}_1 - \left(k_{u1}+k_{u2}\right)\mathrm{co}_{u12}\mathrm{DNA}_{12}\\ &\quad - k_{b3}\mathrm{co}_{b13}\mathrm{co}_{b23}\mathrm{co}_{b123}d\mathrm{DNA}_{12} + k_{u3}\mathrm{co}_{u13}\mathrm{co}_{u23}\mathrm{co}_{u123}\mathrm{DNA}_{123},\end{aligned}$$

$$\begin{aligned}\frac{\mathrm{dDNA}_{23}}{\mathrm{d}t} &= k_{b2}\mathrm{co}_{b23}d\mathrm{DNA}_3 + k_{b3}\mathrm{co}_{b23}d\mathrm{DNA}_2 - \left(k_{u2}+k_{u3}\right)\mathrm{co}_{u23}\mathrm{DNA}_{23}\\ &\quad - k_{b1}\mathrm{co}_{b12}\mathrm{co}_{b13}\mathrm{co}_{b123}d\mathrm{DNA}_{23} + k_{u1}\mathrm{co}_{u12}\mathrm{co}_{u13}\mathrm{co}_{u123}\mathrm{DNA}_{123},\end{aligned}$$

$$\begin{aligned}\frac{\mathrm{dDNA}_{13}}{\mathrm{d}t} &= k_{b1}\mathrm{co}_{b13}d\mathrm{DNA}_3 + k_{b3}\mathrm{co}_{b13}d\mathrm{DNA}_1 - \left(k_{u1}+k_{u3}\right)\mathrm{co}_{u13}d\mathrm{DNA}_{13}\\ &\quad - k_{b2}\mathrm{co}_{b12}\mathrm{co}_{b23}\mathrm{co}_{b123}\mathrm{DNA}_{13} + k_{u2}\mathrm{co}_{u12}\mathrm{co}_{u23}\mathrm{co}_{u123}\mathrm{DNA}_{123},\end{aligned}$$

$$\begin{aligned}\frac{\mathrm{dmRNA}}{\mathrm{d}t} &= \phi_0\left(\mathrm{DNA}_0 + f_1\mathrm{DNA}_1 + f_2\mathrm{DNA}_2 + f_3\mathrm{DNA}_3 + f_{12}\mathrm{DNA}_{12} + f_{23}\mathrm{DNA}_{23} + f_{13}\mathrm{DNA}_{13}\right.\\ &\quad \left.+ f_{123}\mathrm{DNA}_{123}\right) - \gamma_{\mathrm{mRNA}}\mathrm{mRNA},\end{aligned}$$

$$\frac{\mathrm{d}m}{\mathrm{d}t} = \beta\mathrm{mRNA} - 2\alpha_{\mathrm{ass}}m^2 + 2\alpha_{\mathrm{diss}}d - \gamma_m m \text{ and}$$

$$\begin{aligned}\frac{\mathrm{d}d}{\mathrm{d}t} &= -\left(k_{b1}+k_{b2}+k_{b3}\right)d\mathrm{DNA}_0 + k_{u1}\mathrm{DNA}_1 + k_{u2}\mathrm{DNA}_2 + k_{u3}\mathrm{DNA}_3\\ &\quad - \left(\left(k_{b2}\mathrm{co}_{b12} + k_{b3}\mathrm{co}_{b13}\right)d\mathrm{DNA}_1 + \left(k_{b1}\mathrm{co}_{b12} + k_{b3}\mathrm{co}_{b23}\right)d\mathrm{DNA}_2 + \left(k_{b1}\mathrm{co}_{b13} + k_{b2}\mathrm{co}_{b23}\right)d\mathrm{DNA}_3\right)\\ &\quad + \left(k_{u1}+k_{u2}\right)\mathrm{co}_{u12}\mathrm{DNA}_{12} + \left(k_{u2}+k_{u3}\right)\mathrm{co}_{u23}\mathrm{DNA}_{23} + \left(k_{u1}+k_{u3}\right)\mathrm{co}_{u13}\mathrm{DNA}_{13}\\ &\quad - \left(k_{b1}\mathrm{co}_{b12}\mathrm{co}_{b13}\mathrm{DNA}_{23} + k_{b2}\mathrm{co}_{b12}\mathrm{co}_{b23}\mathrm{DNA}_{13} + k_{b3}\mathrm{co}_{b13}\mathrm{co}_{b23}\mathrm{DNA}_{12}\right)\mathrm{co}_{b123}d\\ &\quad + \left(k_{u1}\mathrm{co}_{u12}\mathrm{co}_{u13} + k_{u2}\mathrm{co}_{u12}\mathrm{co}_{u23} + k_{u3}\mathrm{co}_{u13}\mathrm{co}_{u23}\right)\mathrm{co}_{u123}\mathrm{DNA}_{123}\\ &\quad + \alpha_{\mathrm{ass}}m^2 - \alpha_{\mathrm{diss}}d - \gamma_d d,\end{aligned}$$

with $\mathrm{DNA}_{123} = \mathrm{DNA}_{\mathrm{tot}} - \mathrm{DNA}_0 - \mathrm{DNA}_1 - \mathrm{DNA}_2 - \mathrm{DNA}_3 - \mathrm{DNA}_{12} - \mathrm{DNA}_{13} - \mathrm{DNA}_{23}$ because the total amount of DNA is conserved. A summary of all variables and parameters is given in Table A. The quasi steady states of the DNA complexes and dimer are:

$$\mathrm{DNA}_{0,\ \mathrm{qss}} = \frac{\mathrm{DNA}_{\mathrm{tot}}}{\omega_{123}\omega_{12}\omega_{13}\omega_{23}K_1K_2K_3d^3 + (\omega_{12}K_1K_2 + \omega_{23}K_2K_3 + \omega_{13}K_1K_3)\,d^2 + (K_1 + K_2 + K_3)\,d + 1},$$

$$\mathrm{DNA}_{1,\ \mathrm{qss}} = \mathrm{DNA}_{0,\ \mathrm{qss}}K_1d,$$

$$\mathrm{DNA}_{2,\ \mathrm{qss}} = \mathrm{DNA}_{0,\ \mathrm{qss}}K_2d,$$

$$\mathrm{DNA}_{3,\ \mathrm{qss}} = \mathrm{DNA}_{0,\ \mathrm{qss}}K_3d,$$

$$\mathrm{DNA}_{12,\ \mathrm{qss}} = \mathrm{DNA}_{0,\ \mathrm{qss}}\omega_{12}K_1K_2d,$$

$$\mathrm{DNA}_{23,\ \mathrm{qss}} = \mathrm{DNA}_{0,\ \mathrm{qss}}\omega_{23}K_2K_3d,$$

$$\mathrm{DNA}_{13,\ \mathrm{qss}} = \mathrm{DNA}_{0,\ \mathrm{qss}}\omega_{13}K_1K_3d,$$

$$d_{\mathrm{qss}} = \frac{\alpha_{\mathrm{ass}}}{\alpha_{\mathrm{diss}} + \gamma_d}m^2.$$

B Physiological ranges of the parameters

Parameter	Boundaries	Parameter	Boundaries
$\beta(\mathrm{min}^{-1})$	$0.01 - 120$	$f_{\mathrm{activation}}$	$1 - 100$
$\gamma_m(\mathrm{min}^{-1})$	$10^{-3} - 1$	$f_{\mathrm{repression}}$	$0.001 - 1$
$\gamma_{\mathrm{mRNA}}(\mathrm{min}^{-1})$	$10^{-3} - 10$	f	$0.001 - 100$
$\gamma_d(\mathrm{min}^{-1})$	$5 \cdot 10^{-4} - 0.1$	$k_b(\mathrm{min}^{-1})$	$10^{-3} - 10^2$
$\alpha_{\mathrm{ass}}(\mathrm{min}^{-1})$	$10^{-2} - 1$	$k_u(\mathrm{min}^{-1})$	$0.01 - 1000$
$\alpha_{\mathrm{diss}}(\mathrm{min}^{-1})$	$10^{-3} - 10^3$	co_b	$0.05 - 20$
$\phi_0(\mathrm{min}^{-1})$	$10^{-2} - 10$	co_u	$0.05 - 20$

Table B. Physiological ranges for the different parameters [1–4] for a cell with volume $4 - 40$fL (1nM $= 2.4 - 24$molecules)

C Oscillations

C.1 Bifurcation analysis

In order to check if a system oscillates, we first look if we can find a single steady state which is unstable (at least one of the eigenvalues of the Jacobian evaluated in this steady state has a positive real part). Unstable steady states can lead to solutions that oscillate, chaotic behavior or solutions that go to infinity. To be certain the system is oscillating, we do a time series and look for two characteristics of stable oscillations:

1. Stable amplitude: the difference between consecutive maxima in the time series must be less than 1%, the same holds for consecutive minima.

2. Stable period: the difference between to consecutive distances between local maxima must be small.

We want to find the oscillatory regions around a found oscillating solution for every parameter. This means all parameters are kept constant except for one, the parameter of which we determine the bifurcation points. Therefore we use method based on the bisection method for finding roots. To find the left boundary of the oscillating region, we first check whether the system with the considered parameter equal to the left boundary of the physiological range is oscillating. If this is the case, we have found the left boundary of the oscillating region, if not, we know that the bifurcation happens in the interval $[l_1, l_2]$ where we initialize l_1 with the parameter value at the left boundary of the physiological range and l_2 with the parameter value of the oscillating solution. Next we will update the boundaries of the interval around the bifurcation until the interval is considerably small and approximate the bifurcation point by the point that is logarithmically halfway this interval. To update the interval we take the point logarithmically halfway the interval

$$l_m = 10^{\frac{\log_{10}(l_1)+\log_{10}(l_2)}{2}} \tag{1}$$

and check whether it is oscillating. In the case that it is oscillating we update right bound of the interval, $l_2 = l_m$, otherwise the left bound $l_1 = l_m$. The right boundary of the oscillating region is found in a similar way. Finding these two outer solutions does not guarantee that all solutions in the range are oscillating, since other bifurcations might happen in between. Therefore we computed time series for five logarithmically equally spaced samples to see if they were oscillating (large computation time limits the number of solutions that can be tested).

C.2 Oscillatory ranges distributions

In the main paper, the mean values and mean ranges are given for the different parameters. Here different bifurcation ranges are given for different solutions (Figs A, B, C and D). We can see that some parameters can have different values in the physiological range but still be finely tuned, for example β or ϕ_0 for the MDS. For other parameters such as f_i of the 2DS the distribution is bimodal. These characteristics are not represented in the main paper, but are unimportant for a global overview of the oscillating regions. Because there is much variation in the ranges, there is also much variation in the volumes (Fig E).

D Bistability

In order to check if a system is bistable, we look if we can find three steady states of which one is unstable (at least one of the eigenvalues of the Jacobian evaluated in this steady state has a positive real part) and the other two stable (all eigenvalues of the Jacobian evaluated in this steady state have a negative real part).

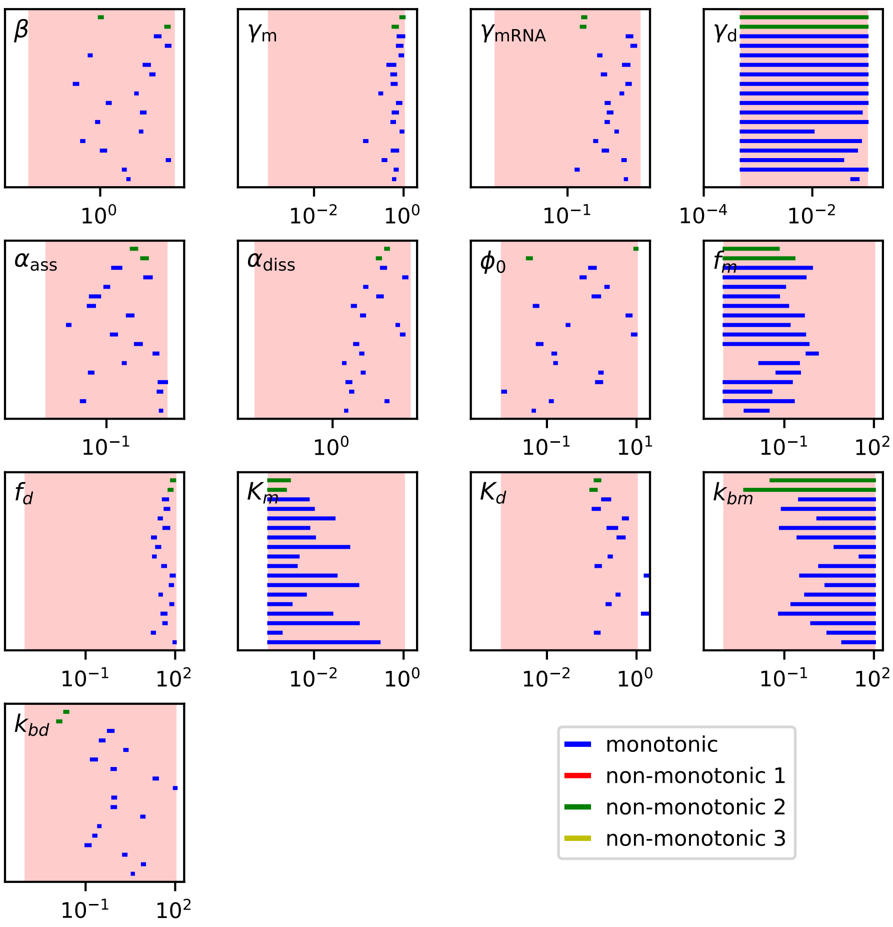

Fig A. Oscillatory ranges of the different parameters for the different solutions of the MDS. The red region represents the physiological range. The axis is logarithmically scaled, the length of the lines thus represent fold ratios. Different lines represent different solutions.

D.1 Quasi steady state approximation

In the assumption of fast binding and unbinding of the dimers to the DNA and fast dimer association and dissociation, we can equal the time derivatives of the DNA complexes and the dimer concentration d to zero and use their quasi steady state approximation in the equations for mRNA and monomer concentration m (given in Section 1):

$$\frac{\mathrm{d}m}{\mathrm{d}t} = \beta \mathrm{mRNA} - \gamma_m m - 2\alpha_{\mathrm{ass}} m^2 + 2\alpha_{\mathrm{diss}} d_{qss}, \tag{2}$$

$$\frac{\mathrm{dmRNA}}{\mathrm{d}t} = \phi_0 \sum_i f_i \mathrm{DNA}_{\mathrm{qss,\ i}} - \gamma_{\mathrm{mRNA}} \mathrm{mRNA} \tag{3}$$

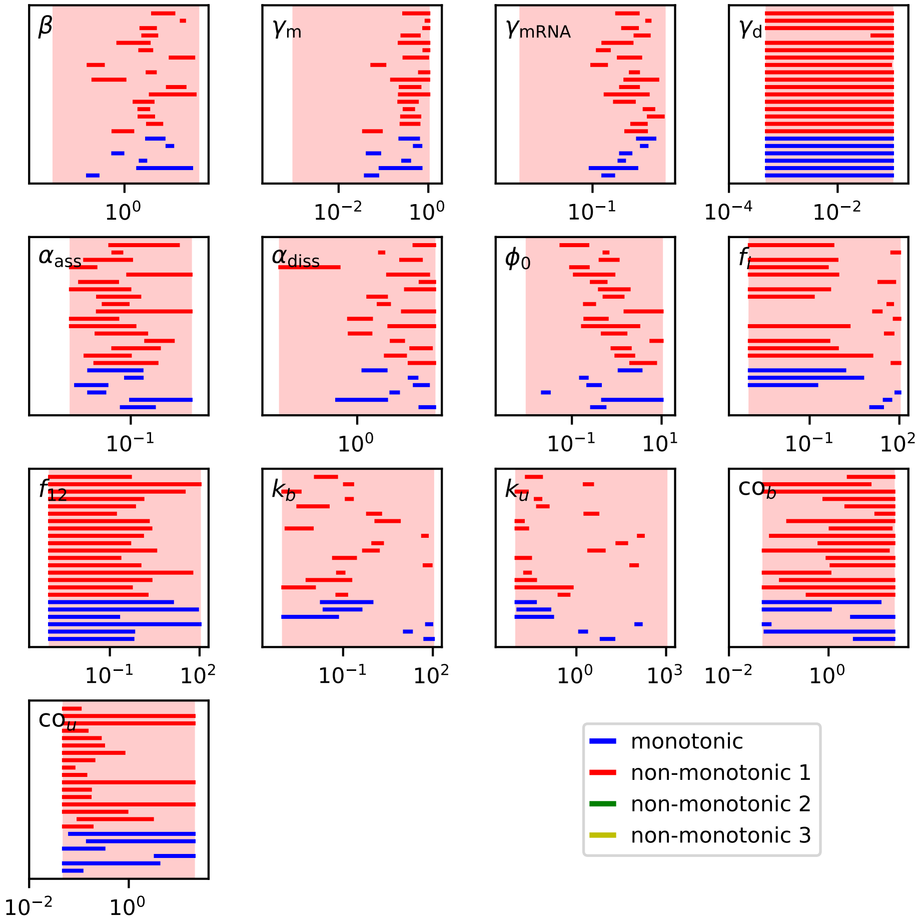

Fig B. Oscillatory ranges of the different parameters for selection of solutions of the 2DS. The red region represents the physiological range. The axis is logarithmically scaled, the length of the lines thus represent fold ratios. Different lines represent different solutions.

with $i \in [0, m, d]$ for the MDS, $i \in [0, 1, 2, 12]$ for the 2DS and $i \in [0, 1, 2, 3, 12, 23, 13, 123]$ for the 3DS. Assuming quasi steady state for the mRNA concentration and fast dissociation of the dimer with respect to degradation of the dimer ($\gamma_d \ll \alpha_{\text{diss}}$), we obtain

$$\frac{\mathrm{d}m}{\mathrm{d}t} = \frac{\beta \phi_0 \mathrm{DNA}_{\text{tot}} f(m,d)}{\gamma_{\text{mRNA}}} - \gamma_m m. \tag{4}$$

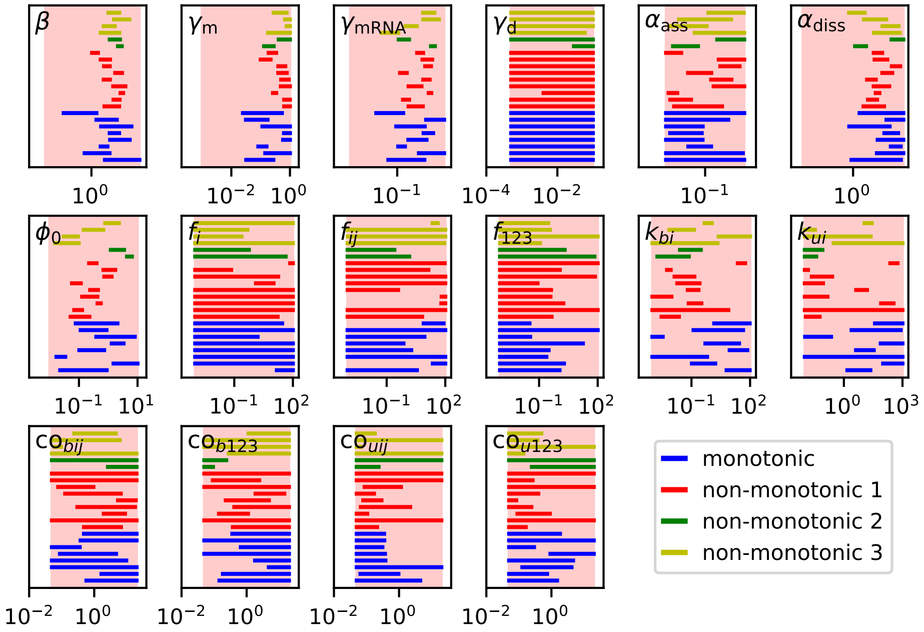

Fig C. Oscillatory ranges of the different parameters for a selection of solutions of the 3DS. The red region represents the physiological range. The axis is logarithmically scaled, the length of the lines thus represent fold ratios. Different lines represent different solutions.

with $f(m,d) = \sum_i f_i \text{DNA}_{\text{qss, i}}/\text{DNA}_{\text{tot}}$ the transcription function. This function depends on the system, using the steady state expression of Section A we obtain

$$f_{MDS}(m,d) = \frac{f_d K_d d + f_m K_m m + 1}{K_d d + K_m m + 1}, \tag{5}$$

$$f_{2DS}(d) = \frac{Ad^2 + Bd + 1}{Cd^2 + Dd + 1}, \tag{6}$$

$$f_{3DS}(d) = \frac{A'd^3 + B'd^2 + C'd + 1}{D'd^3 + E'd^2 + F'd + 1}, \tag{7}$$

with

$$\begin{aligned} C &= K_{d1}K_{d2}\omega, \\ D &= K_{d1} + K_{d2}, \\ D' &= K_{d1}K_{d2}K_{d3}\omega_{12}\omega_{13}\omega_{23}\omega_{123}, \\ E' &= K_{d1}K_{d2}\omega_{12} + K_{d1}K_{d3}\omega_{13} + K_{d2}K_{d3}\omega_{23}, \\ F' &= K_{d1} + K_{d2} + K_{d3} \text{ and} \end{aligned}$$

$$\begin{aligned} A &= f_{12}C, \\ B &= f_1K_{d1} + f_2K_{d2} \\ A' &= f_{123}D, \\ B' &= f_{12}K_{d1}K_{d2}\omega_{12} + f_{13}K_{d1}K_{d3}\omega_{13} \\ &\quad + f_{23}K_{d2}K_{d3}\omega_{23}, \\ C' &= f_1K_{d1} + f_2K_{d2} + f_3K_{d3}. \end{aligned}$$

D.2 Induction time of bistable systems

The induction time is a representation of the time it takes a bistable system to attain the stable high steady state when starting from the unstable intermediate steady state. Deterministically, systems

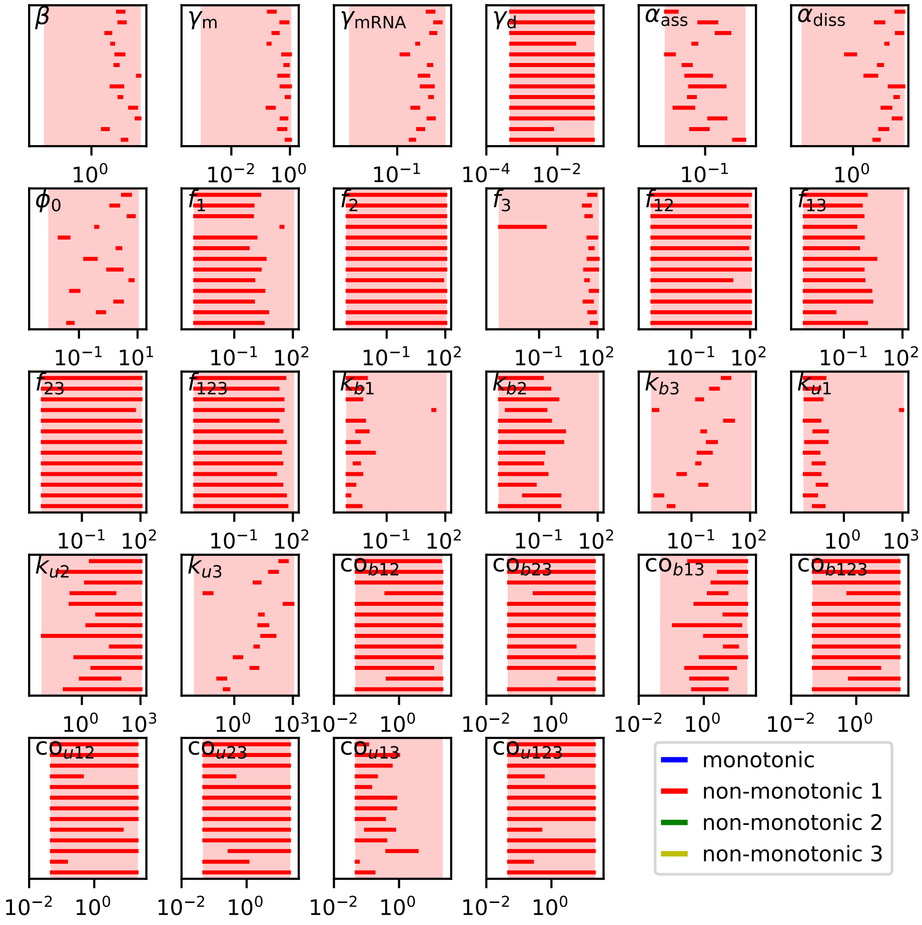

Fig D. Oscillatory ranges of the different parameters for a selection of solutions of the Ss-LrpB. The red region represents the physiological range. The axis is logarithmically scaled, the length of the lines thus represent fold ratios. Different lines represent different solutions.

will remain forever in the unstable intermediate steady state if they are not perturbed, therefore the initial condition is chosen to be a slight perturbation,

$$
\begin{aligned}
s_i = \{&\mathrm{DNA_j} = \mathrm{DNA}_{\mathrm{j},I} \ \forall j \in S, \\
&\mathrm{mRNA} = \mathrm{mRNA}_I, \\
&m = m_I, \\
&d = 1.1 d_I\}
\end{aligned}
$$

where S is $(0, m, d)$ for the MDS, $(0, 1, 2, 12)$ for the 2DS and $(0, 1, 2, 3, 12, 13, 23, 123)$ for the 3DS case and x_H, x_I and x_L are respectively the high, intermediate and low steady state of component x. A deterministic simulation will never reach the high steady state but approach this solution as an asymptote. Therefore the final state is defined by a state which has the dimer concentration higher

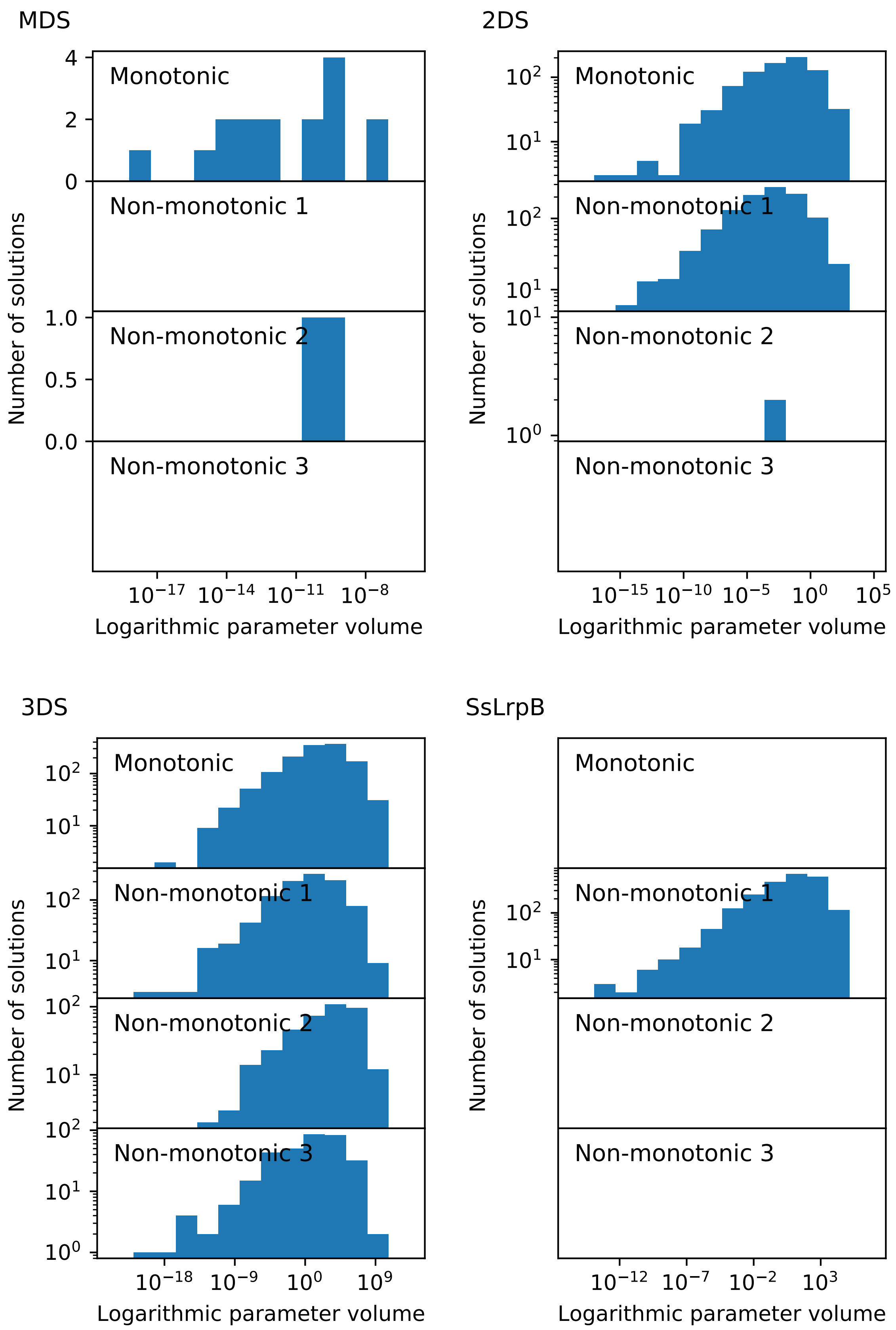

Fig E. Volume distribution for the different toy models.

than 90% of the high steady state,

$$s_f = \{\mathrm{DNA_j}\ \forall j \in S,\\ \mathrm{mRNA},\\ m,\\ d > 0.9 d_H\}.$$

The induction time Δt is thus defined by the time it takes the system to attain a dimer number higher than $0.9d_H$ starting from initial condition s_i.

Simulations show that different choices for K_i, f_i, co_{bi} and co_{ui} – when coefficients A, B, C, D, E and F are fixed– do not influence the induction time. Other free parameters get the values dictated by Table C.

Parameter	Value
γ_m	$0.1\,\mathrm{min}^{-1}$
γ_{mRNA}	$0.01\,\mathrm{min}^{-1}$
γ_d	$0.001\,\mathrm{min}^{-1}$
α_{ass}	$0.01\,\mathrm{min}^{-1}\,\mathrm{molecule}^{-1}$
α_{diss}	$2\,\mathrm{min}^{-1}$
ϕ_0	$1\,\mathrm{min}^{-1}$
k_{bi}	$1\,\mathrm{min}^{-1}$
β	$\left(\gamma_m \gamma_{\mathrm{mRNA}} \sqrt{(\alpha_{\mathrm{diss}} + \gamma_d)/\alpha_{\mathrm{ass}}}\right) / (\gamma \phi_0)$

Table C. Values used for the simulation to measure the induction time of bistable systems

We compare the induction time calculated by an explicit simulation as explained in this section with the approximated induction time $\tilde{\Delta t}$ defined in the main text of the paper (Fig F). As mentioned earlier in this section, the initial condition and final state need to be defined. These will affect the simulated induction time. Depending on the exact shape of the response curve and the parameters, the approximated time is thus an over- or underestimation of the simulated induction time. This explains why there is no one-on-one match of the approximated time and the simulated time.

D.3 Bistability in parameter space

In Fig G in the regions within parameter space providing bistable switches for the different systems are represented. For 2DS and 3DS, a large proportion of parameter space leads to bistability while the MDS needs to be finely tuned to provide this dynamical property. In this figure, we used parameters $A - F$. In order to study the parameters listed in Table A, we selected 100 random solutions in the bistable region and performed a bifurcation analysis. This bifurcation analysis is similar to the one for oscillations, but the boundaries are checked for bistability instead of oscillations. The mean parameter value and mean width of the bistable range is given in Fig H.

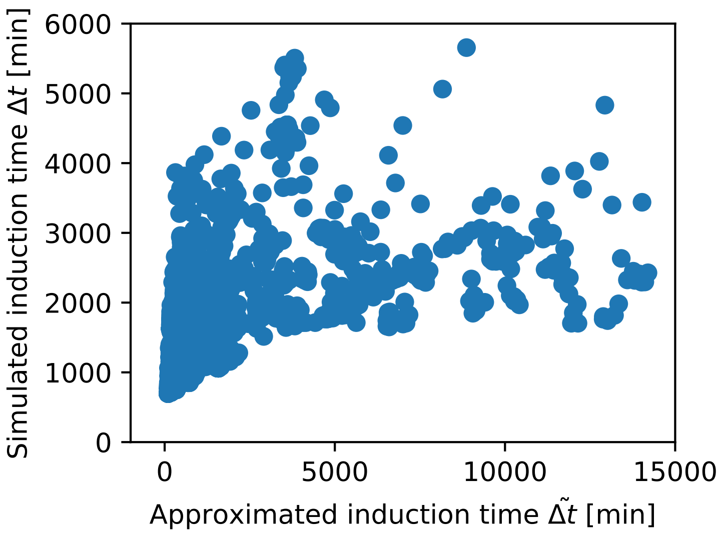

Fig F. Comparison of the approximated and simulated induction time.

E SsLrpB compatibility

In order to be compatible with the natural SsLrpB system, a 3DS needs to have the same values as measured experimentally (Table D) and moreover the response curve needs to be of non-monotonic type 1 with a maximum higher than 2 and a minimum lower than 0.5 [5]. The radius of Sulfolobus solfataricus is around 1 µm, thus the volume can be estimated at 4 fL. A concentration of 1 µM in one cell corresponds therefore to 2400 molecules. Since the non-monotonicity is experimentally shown between 0 and 100 nM [5], we only considered systems up to 1000 molecules (equivalent to 300 nM), i.e. the response curve needed to meet the above constraints on non-monotonicity in the interval from 0 to 1000 dimers, only considering the integer numbers.

The measured parameters of Table D fix parameters, A, D, E and F, which are defined in the main paper, such that only 2 free parameters remain for the bistable switch search : B and C. The former is determined by f_{12}, f_{23} and f_{13} and the latter by f_{123}. For each $f_{13} - f_{123}$ combination, we scanned over the only free parameters f_{12} and f_{23}, for which we get different response curves. These are shown in grey in Fig I. Bistable curves meeting the conditions as explained in the main paper (Eq. 1 of main paper) have a dashed red line and curves meeting the Ss-LrpB criteria have a dashed blue line. For every bistable (red) response curve, different degradation rates are tested (green lines) and the one with the smallest approximated induction time is chosen. A simulation is done with this solution to calculate the real induction time, which is shown by the yellow-green coloring in Fig 9 of the main paper.

References

1. Buchler NE, Gerland U, Hwa T. Nonlinear protein degradation and the function of genetic circuits. Proceedings of the National Academy of Sciences. 2005;102(27):9559–9564. doi:10.1073/pnas.0409553102.

2. Stricker J, Cookson S, Bennett MR, Mather WH, Tsimring LS, Hasty J. A fast, robust and tunable synthetic gene oscillator. Nature. 2008;456(7221):516–519. doi:10.1038/nature07389.

3. Karapetyan S, Buchler NE. Role of DNA binding sites and slow unbinding kinetics in titration-based oscillators. Physical Review E. 2015;92(6). doi:10.1103/PhysRevE.92.062712.

Parameter	value	
$K_{d1} = k_{b1}/k_{u1}$	$73.5\,\mu\text{M}^{-1}$	3.1×10^{-2} molecule^{-1}
$K_{d2} = k_{b2}/k_{u2}$	$0.7\,\mu\text{M}^{-1}$	2.9×10^{-4} molecule^{-1}
$K_{d3} = k_{b3}/k_{u3}$	$49.1\,\mu\text{M}^{-1}$	2.0×10^{-2} molecule^{-1}
$\omega_{12} = \text{co}_{b12}/\text{co}_{u12}$	4.1	
$\omega_{13} = \text{co}_{b13}/\text{co}_{u13}$	2.1	
$\omega_{23} = \text{co}_{b23}/\text{co}_{u23}$	7.9	
$\omega_{123} = \text{co}_{b123}/\text{co}_{u123}$	3.1	

Table D. Measured values for the Ss-LrpB system

4. Zavala E, Marquez-Lago T. Delays Induce Novel Stochastic Effects in Negative Feedback Gene Circuits. Biophysical Journal. 2014;106(2):467–478. doi:10.1016/j.bpj.2013.12.010.

5. Peeters E, Peixeiro N, Sezonov G. Cis-regulatory logic in archaeal transcription. Biochemical Society Transactions. 2013;41(1):326–331. doi:10.1042/BST20120312.

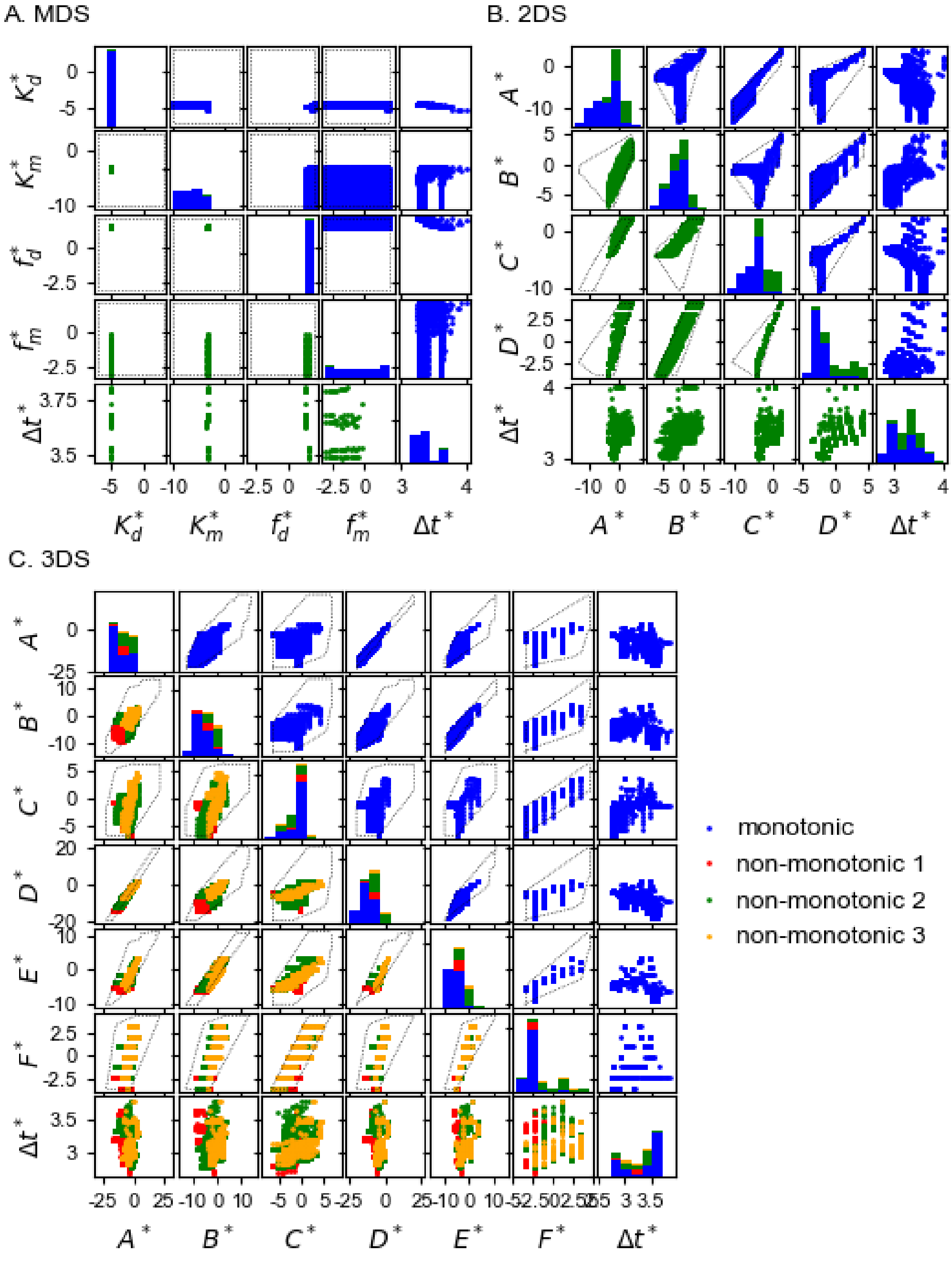

Fig G. Parameter distribution of bistable solutions for the different toy models. Bistable solutions are shown as a function of the logarithm of the parameters dictating the response curve and the logarithm of the induction time ($X^* = \log_{10}(X)$ with $X \in \{K_d, K_m, f_d, f_m, \Delta t, A, B, C, D, E, F\}$). Monotonic solutions are shown in the upper triangles (blue solutions). Non-monotonic solutions are shown in the lower triangles (red, green and orange). Gray dashed lines indicate physiological ranges.

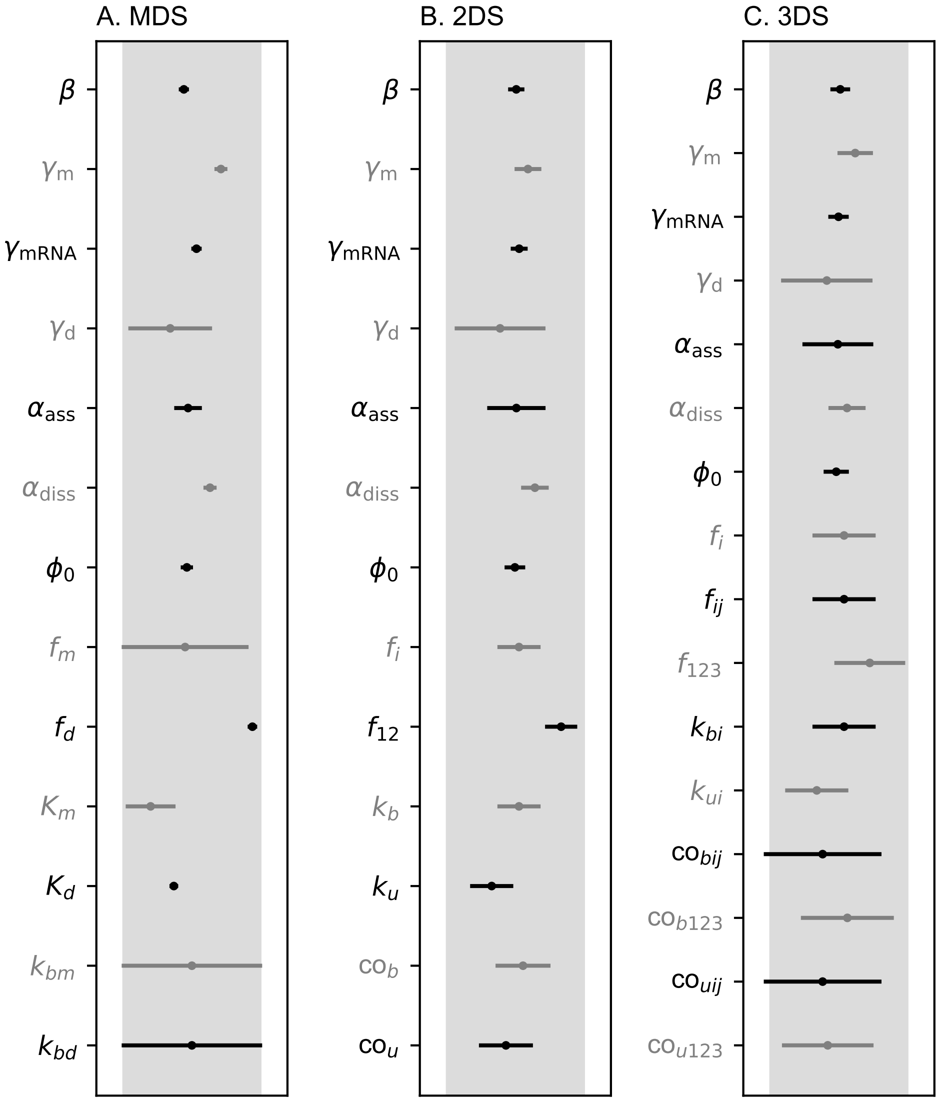

Fig H. Bistable solutions for the different toy models.
The shaded region represents the physiological range. The black and gray lines represent the mean bistable ranges for the different parameters. The axis is logarithmically scaled, the length of the lines thus represent fold ratios. The line for each parameter is scaled according to the physiological range of this parameter. Ranges are very small for the MDS and become wider for the 2DS and 3DS.

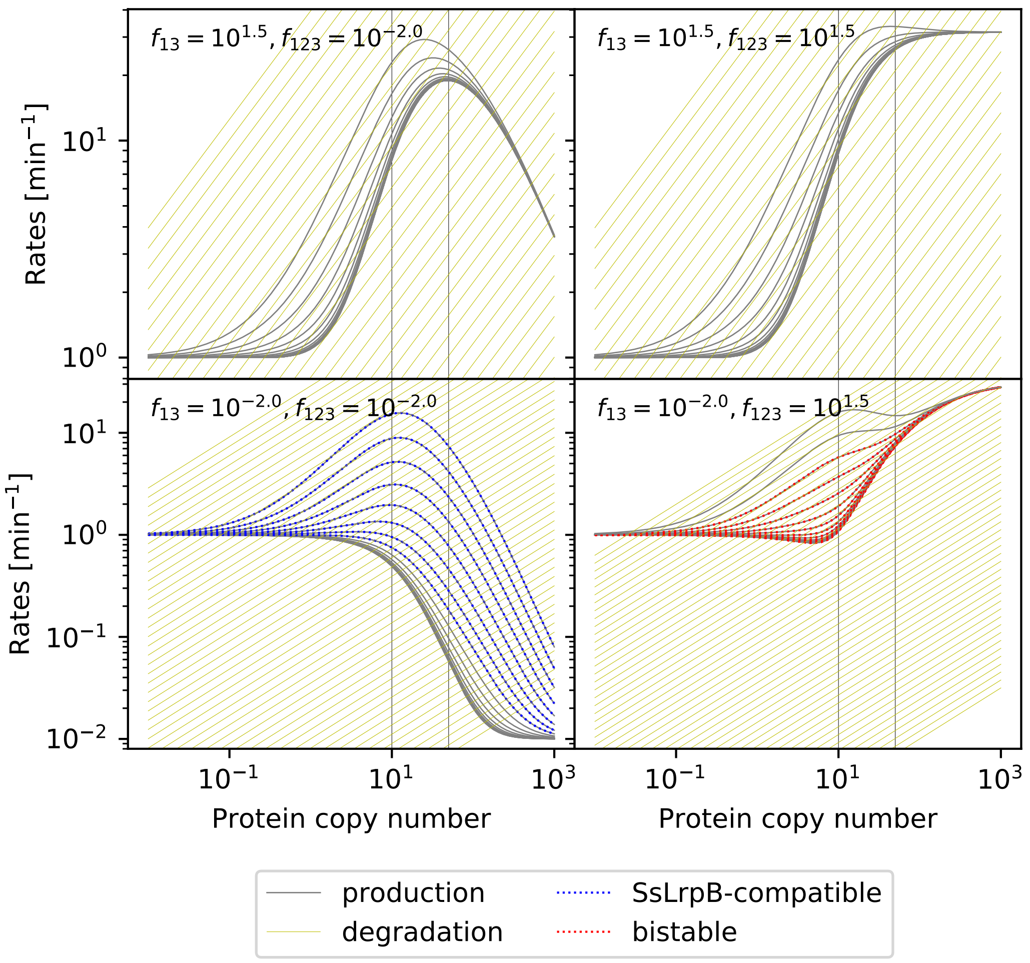

Fig I. Rates for different $f_{13} - f_{123}$ combinations.
